# The Autism Biomarkers Consortium for Clinical Trials: evaluation of a battery of candidate eye-tracking biomarkers for use in autism clinical trials

**DOI:** 10.1186/s13229-021-00482-2

**Published:** 2022-03-21

**Authors:** Frederick Shic, Adam J. Naples, Erin C. Barney, Shou An Chang, Beibin Li, Takumi McAllister, Minah Kim, Kelsey J. Dommer, Simone Hasselmo, Adham Atyabi, Quan Wang, Gerhard Helleman, April R. Levin, Helen Seow, Raphael Bernier, Katarzyna Charwaska, Geraldine Dawson, James Dziura, Susan Faja, Shafali Spurling Jeste, Scott P. Johnson, Michael Murias, Charles A. Nelson, Maura Sabatos-DeVito, Damla Senturk, Catherine A. Sugar, Sara J. Webb, James C. McPartland

**Affiliations:** 1 Center for Child Health, Behavior, and Development, Seattle Children’s Research Institute, 1920 Terry Ave, Seattle, WA 98101 USA; 2 Department of General Pediatrics, University of Washington School of Medicine, Seattle, WA USA; 3 Yale Child Study Center, Yale University School of Medicine, 230 South Frontage Road, New Haven, CT 06520 USA; 4 Paul G. Allen School of Computer Science and Engineering, University of Washington, Seattle, WA USA; 5 Department of Computer Science, University of Colorado - Colorado Springs, Colorado Springs, CO USA; 6 Department of Biostatistics, University of California Los Angeles, Los Angeles, CA USA; 7 Department of Neurology, Boston Children’s Hospital, Boston, MA USA; 8 Harvard Medical School, Boston, MA USA; 9 Department of Psychiatry and Behavioral Science, University of Washington School of Medicine, Seattle, WA USA; 10 Duke Center for Autism and Brain Development, Duke University, Durham, NC USA; 11 Emergency Medicine, Yale University School of Medicine, New Haven, CT USA; 12 Department of Pediatrics, Boston Children’s Hospital, Boston, MA USA; 13 Present Address: Division of Neurology, Department of Pediatrics, Children’s Hospital Los Angeles, Los Angeles, CA USA; 14 Department of Psychology, University of California Los Angeles, Los Angeles, CA USA; 15 Institute for Innovations in Developmental Sciences, Northwestern University, Chicago, IL USA; 16 Graduate School of Education, Harvard University, Boston, MA USA; 17 Department of Psychology, Yale University, 2 Hillhouse Ave, New Haven, CT 06520 USA; 18 Department of Psychology, University of Virginia, 102 Gilmer Hall, P.O. Box 400400, Charlottesville, VA 22904 USA; 19 Department of Biostatistics, School of Public Health, University of Alabama at Birmingham, Birmingham, AL USA

**Keywords:** Autism spectrum disorder, Biomarkers, Eye tracking, Visual attention, Face processing, Biological motion, Gaze pattern

## Abstract

**Background:**

Eye tracking (ET) is a powerful methodology for studying attentional processes through quantification of eye movements. The precision, usability, and cost-effectiveness of ET render it a promising platform for developing biomarkers for use in clinical trials for autism spectrum disorder (ASD).

**Methods:**

The Autism Biomarkers Consortium for Clinical Trials conducted a multisite, observational study of 6–11-year-old children with ASD (*n* = 280) and typical development (TD, *n* = 119). The ET battery included: Activity Monitoring, Social Interactive, Static Social Scenes, Biological Motion Preference, and Pupillary Light Reflex tasks. A priori, gaze to faces in Activity Monitoring, Social Interactive, and Static Social Scenes tasks were aggregated into an Oculomotor Index of Gaze to Human Faces (OMI) as the primary outcome measure. This work reports on fundamental biomarker properties (data acquisition rates, construct validity, six-week stability, group discrimination, and clinical relationships) derived from these assays that serve as a base for subsequent development of clinical trial biomarker applications.

**Results:**

All tasks exhibited excellent acquisition rates, met expectations for construct validity, had moderate or high six-week stabilities, and highlighted subsets of the ASD group with distinct biomarker performance. Within ASD, higher OMI was associated with increased memory for faces, decreased autism symptom severity, and higher verbal IQ and pragmatic communication skills.

**Limitations:**

No specific interventions were administered in this study, limiting information about how ET biomarkers track or predict outcomes in response to treatment. This study did not consider co-occurrence of psychiatric conditions nor specificity in comparison with non-ASD special populations, therefore limiting our understanding of the applicability of outcomes to specific clinical contexts-of-use. Research-grade protocols and equipment were used; further studies are needed to explore deployment in less standardized contexts.

**Conclusions:**

All ET tasks met expectations regarding biomarker properties, with strongest performance for tasks associated with attention to human faces and weakest performance associated with biological motion preference. Based on these data, the OMI has been accepted to the FDA’s Biomarker Qualification program, providing a path for advancing efforts to develop biomarkers for use in clinical trials.

**Supplementary Information:**

The online version contains supplementary material available at 10.1186/s13229-021-00482-2.

## Introduction

Autism spectrum disorder (ASD) is associated with social communication difficulties, the presence of restricted patterns of behaviors, and atypical response to sensory information [1]. ASD is extremely heterogeneous, with extensive variation across individuals in social, cognitive, regulatory, and attentional phenotypes. Progress in developing interventions for ASD has been hindered by a lack of measures that can, within this heterogeneity, provide objective quantification of intrinsic features of ASD with sensitivity, reliability, and mechanistic relationship to core symptoms or intervention response. Biomarkers offer promise to address this need in ASD.

A biomarker is “a defined characteristic that is measured as an indicator of normal biological processes, pathogenic processes, or biological responses to an exposure or intervention” [2]. Biomarkers may quantify performance relevant to specific functional processes [3] and differ from clinical outcome assessments by virtue of focus on objective quantifiability and underlying mechanism. However, currently there exists no widely-accepted biomarkers established with sufficient rigor for guiding clinical practice or for broad use in clinical trials for ASD [for recent discussions, see 4, 5]. One challenge is the extensive infrastructure, spanning methodological, clinical, and trial management expertise, that is often required in order to establish a biomarker’s analytical validity. Acceleration of the clinical trial pipeline through biomarker development and qualification, an area of concerted focus for over 15 years [6, 7], may benefit from the design and evaluation of biomarker primitives with applications to multiple downstream clinical applications.

Social attention is a key functional process relevant to biomarker research in ASD [8]. Across a variety of studies, experimental modalities, and tasks, individuals with ASD exhibit altered attention to social information compared to non-ASD controls [e.g. 9–11; review 12, 13]. ET offers insight into social attention by allowing for the precise moment-by-moment quantification of the gaze patterns of individuals as they visually process social information. Because ET is safe, noninvasive, scalable, and easily tolerated by participants from infancy through adulthood and across a wide range of function including significant cognitive impairment [14], it offers a powerful approach for the identification and development of social attentional biomarkers in heterogeneous conditions such as ASD.

Like many biomarker technologies, ET-based biomarkers for ASD could potentially advance various contexts of use, e.g., as diagnostic, predictive, prognostic, or response biomarkers [15, 16]. Recent work has suggested that ET biomarkers may associate with clinical assessments [17, 18], response to behavioral intervention [19], and administration of novel pharmacological compounds [20, 21]. ET biomarkers additionally may serve as diagnostic enrichment biomarkers [22] to decrease variability in a study population, permitting more efficient evaluation of intervention in smaller homogeneous samples.

Across contexts of use, biomarkers must exhibit specific properties as a requirement for practical utility. For biomarker deployment in clinical trials, minimum requirements are that the biomarkers evidence construct validity, feasibility in data acquisition, and reliability. The Autism Biomarkers Consortium for Clinical Trials (ABC-CT) [23] was designed to develop and validate these aspects of biomarker performance in children with ASD, addressing limitations in currently available studies, specifically small sample sizes and heterogenous acquisition and analytic methodologies [24]. From a candidate set of nine ET biomarkers (originally selected based on a review of extant eye-tracking paradigms demonstrating robust findings across multiple studies or in large samples of children with ASD prior to the inception of project funding), five ET tasks were selected for inclusion based on construct validity, evidence of ASD-control differences, and relation to ASD symptoms in an initial Feasibility Study prior to the Main Study reported here (see [25] for additional details regarding ET biomarker selection). Four of these tasks focused on social-attentional constructs and included: (1) Activity Monitoring (ActivityMonitoring), depicting videos of two adults playing with toys; (2) the Social Interactive (SocialInteractive) task, videos of two children engaged in parallel and joint play; (3) Static Social Scenes (StaticScenes), images depicting varied naturalistic scenes involving people; and (4) Biological Motion Preference (Biomotion), point-light display videos of biological motion versus non-biological control stimuli shown side-by-side. A fifth task, (5) Pupillary Light Reflex (PLR), was included in the ET battery as a measure associated with autonomic nervous system function and measured pupillary constriction in response to a light flash.

A composite variable representing gaze to faces across three tasks (ActivityMonitoring, SocialInteractive, StaticScenes), the Oculomotor Index of Gaze to Human Faces (OMI), was developed a priori (see Supplemental Information) based on preliminary data and served as the overall main outcome measure for the ET battery. Additional primary and secondary variables for each individual task were also pre-specified. Data were acquired and evaluated using stringent and rigorous manualized protocols with evaluation focused on metrics of biomarker viability in terms of (1) feasibility of acquisition, as measured by acquisition rates; (2) construct validity, as demonstrated by expected within-subject task performance in typically developing children; (3) stability across two timepoints separated by six weeks; (4) discrimination between ASD and TD groups as a means of illuminating regimes of atypical performance in ASD; and (5) association with clinical and behavioral phenotypic characteristics. These specific properties were selected for evaluation in order to assess fundamental psychometric properties of examined biomarkers that would be necessary for understanding their applicability and general usability for clinical trials in ASD. See [25] for further details regarding the protocol and analytical design considerations.

The objective of this work was to pair rigorous methodology and a large, well-characterized sample for the purpose of assessing early-stage viability of these markers for use in future biomarker applications for clinical trials. Toward this goal, this work seeks to characterize performance of ET biomarkers across fundamental evaluative dimensions so as to provide a template for ongoing biomarker development and deployment as well as to speak to their applicability for future, specific contexts-of-use.

## Methods and materials

### Autism Biomarkers Consortium for Clinical Trials (ABC-CT) protocol

The first ABC-CT study was a five-site observational study involving clinician, caregiver, and lab-based measures as well as a battery of electroencephalography (EEG) and ET tasks. Participants were school-age children with ASD or typical development (TD) assessed across three timepoints: Time 1 (T1), Time 2 (T2: T1 + 6 weeks), and Time 3 (T3: T1 + 24 weeks), with each timepoint conducted over two days. ET tasks were administered on both days at each timepoint. This report focused on data from T1 and T2, as the six-week span between the two timepoints approximates the duration of many clinical trials and is relevant to understanding short term stability. T3 data are being analyzed elsewhere in the context of longer-term developmental change and change in clinical status.

Informed consent/assent was obtained from all guardians and participants after procedures were fully explained and the opportunity to ask questions offered. The protocol was approved and overseen by a central IRB at Yale University.

An overview of the ABC-CT history and protocol is available in [23], with data acquisition and quality control details in [25]. More extensive protocol, participant, and ET methodological details are provided in Supplemental Information. Study data are available in [26].

### Participant characteristics

Participants were children 6;0 to 11;6 years old at T1, an age range selected to constrain age-related developmental heterogeneity and increase likelihood of successful biomarker data acquisition [23]. Children in the ASD group (*n* = 280) met DSM-5 diagnostic criteria for ASD [1] based on gold-standard research diagnostic criteria with the ADOS-2 and the ADI-R and had full scale IQ between 60 and 150. TD children (*n* = 119) were screened for the presence of ASD, emotional and behavioral disorders (based on [27] and medical history), and had full scale IQs between 80 and 150. Exclusions for both groups included genetic or neurological conditions, or sensory challenges that would impact protocol completion. In the ASD group, medications were stable for 8 weeks prior to enrollment. See Supplemental Information for additional inclusion, exclusion, and assessment details. Groups did not differ by age (*t* = 0.199, *p* = 0.843) nor sex^1^ (Χ^2^ = 2.19, *p* = 0.139) but differed in diagnostic and clinical characterization (Table 1). Patterns of results were unchanged when considering subsets of participants with valid data for each ET biomarker (Additional file 1: Tables S1ab).

**Table 1:** Participant characteristics. Mean and standard deviation are presented for clinical assessments for the full sample at T1. For characterization associated with subsets completing ET tasks, see Additional file 1: Tables S1ab. For clinical variable descriptions, see Additional file 1: Table S2

Time 1 Demographics	ASD	TD	Assessment [*M* (SD)]	ASD	TD
*Participant*			Full Scale IQ	96.58 (18.11)	115.12 (12.55)
*N*	280	119	Verbal IQ	95.95 (20.69)	116.27 (11.22)
Sex [*N* Males: *N* Females]	215: 65	83: 36	Nonverbal IQ	97.52 (16.91)	112.18 (14.05)
%Male	76.8%	69.7%	ADOS CSS	7.65 (1.77)	1.58 (0.87)
Age in years [*M* (SD)]	8.55 (1.64)	8.51 (1.61)	ADOS SA	7.34 (1.79)	1.91 (1.34)
*Participant Race [N (%)]*			ADOS RRB	8.05 (1.73)	3.04 (2.48)
White	190 (67.9%)	98 (82.4%)	VABS3 ABC	73.37 (11.14)	102.74 (9.84)
American Indian/Alaskan Native	2 (0.7%)	0 (0%)	VABS3 Soc SS	69.89 (16.15)	104.55 (9.23)
Black/African American	22 (7.9%)	4 (3.4%)	VABS3 Com SS	76.44 (15.07)	103.44 (9.16)
Asian	15 (5.4%)	2 (1.7%)	SRS-2 Total	73.54 (10.92)	42.57 (4.66)
Mixed race	45 (16.1%)	14 (11.8%)	SRS-2 SCI T	72.65 (10.83)	42.47 (5.05)
Other	6 (2.1%)	1 (0.8%)	SRS-2 RIRB T	73.76 (12.18)	43.97 (3.72)
*Participant Ethnicity [N (%)]*			PDDBI Soc App T	54.21 (9.3)	69.83 (3.04)
Hispanic	52 (18.6%)	8 (6.7%)	PDDBI REPRIT T	49.6 (11.52)	28.03 (2.61)
Non-Hispanic	228 (81.4%)	111 (93.3%)	Face Memory SS	7.86 (3.67)	10.53 (3.49)

ADOS CSS — Autism Diagnostic Observation Schedule calibrated severity score (comparison score); ADOS SA — social affect comparison score; ADOS RRB — restricted interests and repetitive behavior comparison score; VABS3 ABC — Vineland Adaptive Behavior Scales adaptive behavior composite standard score; VABS3 Soc SS — socialization standard score; VABS3 Com SS — communication standard score; SRS-2 Total —Social Responsiveness Scale total T-score; SRS-2 SCI T —social communication and interaction T-score; SRS-2 RIRB T — restricted interest and repetitive behavior T-score; PDDBI Soc App T — Pervasive Developmental Disorders Behavior Inventory Social Approach Behaviors T-score; PDDBI REPRIT T — Repetitive, Ritualistic, and Pragmatic Problems Composite T-score; Face Memory SS — NEPSY memory for faces subtask score

### Data acquisition

ET data acquisition was stringently standardized [25], with all sites achieving and maintaining protocol fidelity through rigorous training, manualization, and quality control procedures overseen by the Data Acquisition and Analysis Core (DAAC) of the ABC-CT. Manuals (see Supplemental Information) are available upon request.

### Equipment

Sites used SR Research Eyelink 1000 Plus binocular remote eye trackers operating at 500 Hz. Stimuli were presented on 24″ 1920 × 1200 pixel 60 Hz monitors and controlled via identically configured presentation computers using Neurobehavioral Systems Presentation v18.1. Video cameras recorded the face and upper torso of the child and were multiplexed with video feeds from the ET control (host) computer and the presentation screen for subsequent behavioral review and quality assurance. See [25] for additional equipment details.

### Protocol

ET sessions began with children seated (eye-to-monitor distance: 65 cm) in front of the stimulus presentation monitor. No head supports/restraints were used. A child-appropriate movie was played to capture the child’s attention, followed by a 5-point ET calibration procedure, and then administration of ET tasks.

Site behavioral assistants added supplemental verbal directions (e.g., “Sit back”, “Talk later”, “Watch TV”) and behavioral supports appropriate to the cognitive level and behavioral needs of children.

ET sessions were conducted on both days of each timepoint, with each session lasting approximately 14.5 min (involving 9.7 min/54 trials of experiments; see Additional file 1: Table S3 for experimental task administration details). Trials from ET tasks were interleaved in blocks to reduce fatigue and optimize child engagement. Validation targets were periodically administered to facilitate error estimation and scanpath recalibration. Task order was counterbalanced across participants.

### Acquisition metrics, quality control, and derived variables

Subsequent to transfer of data from sites to the ABC-CT Data Coordinating Core, acquired ET data were processed centrally by the DAAC to extract acquisition metrics and derived variables.

Trial validity criteria for ActivityMonitoring, SocialInteractive, StaticScenes, and Biomotion tasks were percent of acquired ET data relative to stimulus presentation time (%Valid Data) ≥ 50% and calibration error (Cal Error) ≤ 2.5° (visual degrees, 1° = 42 pixels). For PLR, additional criteria were imposed to ensure rigor of latency and constriction size estimates.

Data from an ET session (single day) were invalidated if experimental counterbalancing errors, technical malfunctions, or non-standardized verbal cues (e.g., specific direction of attention to the stimuli) occurred. Data from an ET timepoint (both days) were invalidated if fewer than 25% of trials were valid (%Valid Trials). The OMI biomarker (made up of ActivityMonitoring, SocialInteractive, and StaticScenes tasks) was considered valid only if all constituent sub-tasks (ActivityMonitoring, SocialInteractive, and StaticScenes) were valid. Aggregated acquisition metrics at the task-level were: %Valid Data, Cal Error, and %Valid Trials.

Derived measures for each individual at each timepoint were averaged over all valid trials for that task. OMI, ActivityMonitoring, SocialInteractive, StaticScenes, and Biomotion involved region-of-interest (ROI) analysis (Additional file 1: Figure S1), where presented scenes were divided into zones associated with semantic labels and the proportion of valid gaze data within those zones calculated (e.g., %Face for percentage of time spent looking at faces). For PLR, latency and relative pupil constriction were computed as in [28].

All quality control (QC) criteria and derived variable definitions were formulated before ABC-CT main study enrollment and maintained throughout the entirety of the study. See Supplementary Information for additional details regarding QC, acquisition metrics, derived variables, and pre-hypothesized effects.

### Experimental tasks

**Fig. 1:**
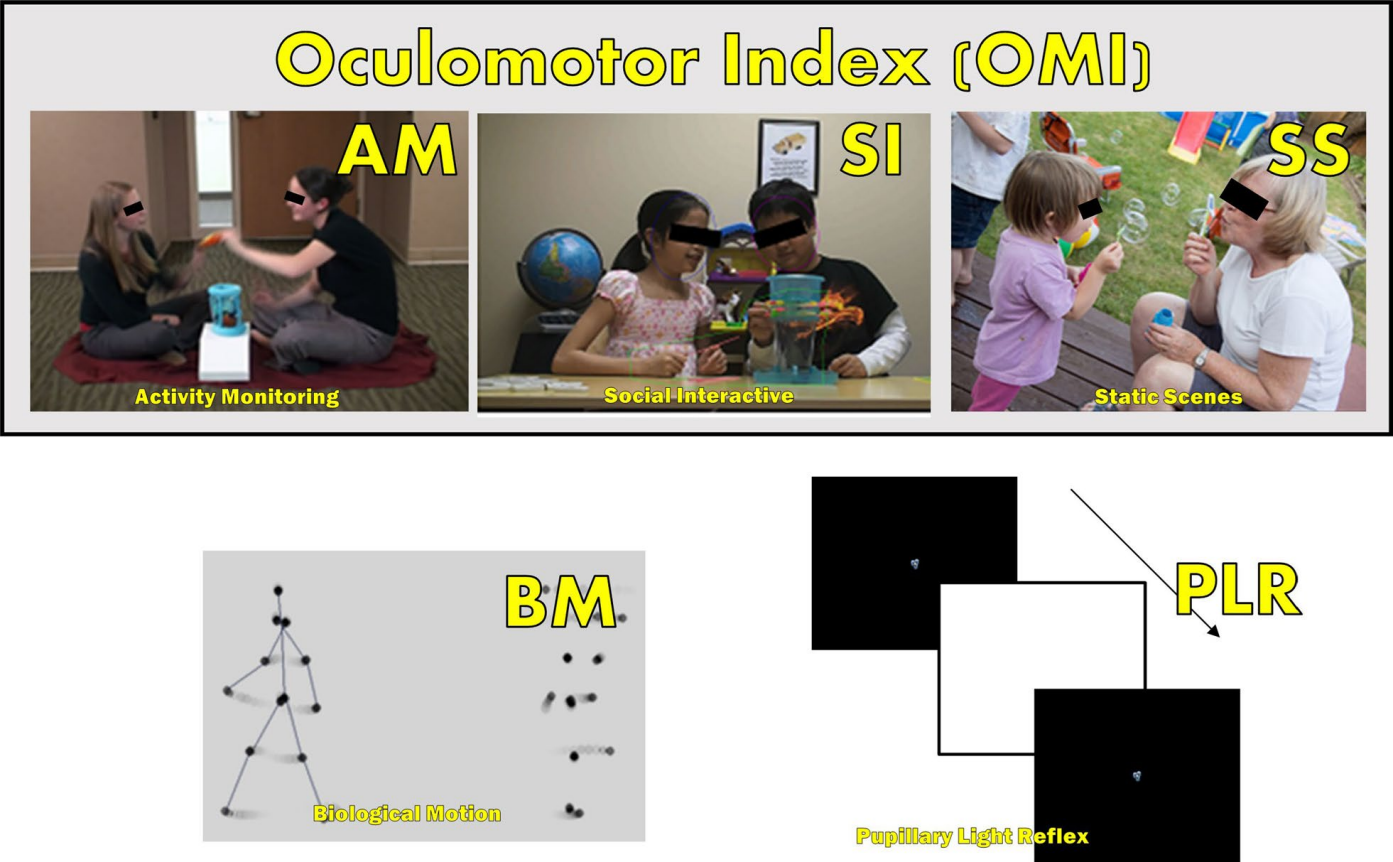
Experimental Tasks. (*Top row*) Tasks comprising the Oculomotor Index of Gaze to Human Faces (OMI): ActivityMonitoring (AM, videos depicting two actors engaged in a shared activity), SocialInteractive Scenes (SI, videos depicting two children involved in interactive and parallel play activities), and StaticScenes (SS, Social Static Scene images showing everyday scenes involving social interactions). (*Bottom row*) Biomotion (BM, Biological Motion preferential looking videos with point-light displays of human actions paired with non-human control conditions. Lines in human figure added for illustrative purposes only), and Pupillary Light Reflex task (PLR, images depict frames in the video sequence including the bright screen flash)

Five experimental ET tasks were administered (Fig. 1). Based on preliminary findings from the ABC-CT Feasibility Study [25], conducted prior to the main study reported here, an additional biomarker, the Oculomotor Index of Gaze to Human Faces (OMI), was constructed as the average of %Face from ActivityMonitoring, SocialInteractive, and StaticScenes tasks. See Additional file 1: Table S3 and Supplementary Information for details regarding experimental tasks including OMI derivation (Additional file 1: Tables S4-5).

### Activity monitoring (activitymonitoring)

This task [29, 30] showed interleaved eight trials of static images (10 s each) and eight trials of dynamic videos (20 s each) of two actresses playing with children’s toys. During static image trials, a wordless soundtrack was played. During video trials, the actresses spoke in child-friendly language and directed their eyes to each other (mutual gaze) or the joint activity (activity gaze). The primary dependent variable was percentage of time spent looking at the heads and faces of the actresses (%Face), relative to the amount of validly acquired ET data during a trial. Secondary variables included percentage of valid time spent looking at actress activities (%Activity).

### Social interactive task (socialinteractive)

This task [31] showed silent 15-s videos of two school-aged children engaged in parallel (11 trials) or cooperative play (11 trials) with toys. The primary dependent variable was percentage of valid time spent looking at heads and faces of actors (%Face). Secondary variables included percentage of valid time spent looking at any part of the actors (%Social: sum of face, body, and activity regions).

### Static social scenes task (staticscenes)

This task showed, for 20-s each, six photographs of solitary and social interactions of children or of children and adults [32]. It was repeated on each day of each timepoint, with images flipped horizontally on the second day. Like the SocialInteractive task, the primary variable was %Face, and secondary %Social.

### Oculomotor index of gaze to human faces (OMI)

A principal component analysis of ET derived variable data from the Feasibility stage of the ABC-CT study (see 23) revealed a primary component dominated by %Face variables from ActivityMonitoring, SocialInteractive, and StaticScenes tasks. As the weights for all of these variables were comparable, we created the OMI biomarker as a composite score averaging ActivityMonitoring, SocialInteractive, and StaticScenes %Face with equal weights.

### Biological motion preference task (biomotion)

The Biological Motion Preference task involved 40 trials of soundless point light displays of human biological motion side-by-side with a non-biological motion control based on [33]. Human biological motion included primitive motor, affective, communicative, tool-oriented, or goal-oriented movements from [34]. Control conditions were either rotating or scrambled point light displays. The primary variable was biological motion preference percentage (%Bio, time looking at biological motion divided by time looking at biological motion or control). Secondary variables included biological motion preference from affective stimuli (%BioAffect).

### Pupillary light reflex task (PLR)

The Pupillary Light Reflex task included 18 trials of a dark screen with a small, 0.7 degree animation at the center, then a flash of white for four frames, followed by the return of the dark screen and central animation [28]. A sound effect accompanied the animation throughout each trial. The primary variable was latency to minimum pupil size acceleration (Latency). Secondary variables included relative pupil constriction (Constrict) [28, 35].

### Analytic plan

Analyses were pre-specified as highlighted in [23, 25]. Notably, examination of distributional characteristics of biomarker outputs [25] did not reveal statistical pathologies that would interfere with analytical interpretation. Nonetheless, ANOVA methods used heteroskedastic consistent covariance matrices to accommodate unequal group variances; correlations relied upon Spearman rank correlation coefficients for robustness against potential leverage effects due to outliers or severe non-normality. See Supplemental Information for additional details on correlation method rationale.

As a primarily descriptive study, no controls for multiple comparisons were enacted. However, we note that hypotheses for primary analytical aims were pre-specified; secondary analyses are presented primarily in Supplemental information.

### Acquisition

For each ET biomarker, we examined rates of data acquisition (percentage of children generating any data) and data validity (percentage of children whose data passed all quality control criteria) (Tables 2, S6ab). We considered > 70% data validity in both ASD and TD groups to index suitability for clinical trials based on data acquisition rates reported in prior published experimental studies, consultation with statistical and biomarker-domain experts, and consensus across project stakeholders and external reviewers. Diagnostic group and potential site differences in acquisition rates were assessed with chi-square tests. Differences in acquisition metrics (%Valid Trials, %Valid Data, and Cal Error) were assessed with univariate ANOVA (Additional file 1: Table S7). Relationships among acquisition metrics and child characteristics were assessed using Spearman’s rank correlation (Additional file 1: Table S8). Analyses were conducted both unadjusted and adjusted for age, IQ, and site.

**Table 2:** Biomarker properties. For extended data see Supplemental Tables.

	OMI	AM	SI	SS	BM	PLR
*Signal Acquisition and Validity at T1*						
ASD						
Acquisition valid	279 (99%)	280 (100%)	280 (100%)	279 (100%)	280 (100%)	278 (99%)
Signal valid	272 (97%)	280 (100%)	276 (99%)	273 (98%)	277 (99%)	266 (95%)
Site differences	*X*^2^ = 6.6, *p* = .16	–	*X*^2^ = 1.0, *p* = .90	*X*^2^ = 7.6, *p* = .11	*X*^2^ = 5.2, *p* = .27	*X*^2^ = 16.1, *p* < .01
TD						
Acquisition valid	119 (100%)	119 (100%)	119 (100%)	119 (100%)	119 (100%)	119 (100%)
Signal valid	119 (100%)	119 (100%)	119 (100%)	119 (100%)	119 (100%)	117 (98%)
Site differences	–	–	–	–	–	*X*^2^ = 3.0, *p* = .56

Task	OMI	AM	SI	SS	BM	PLR
*Construct validity for each ET task based on TD participants at T1*						
Construct	Face preference	Face preference	Face preference	Face preference	Biomotion preference	Pupillary light reflex
Test	All sub-tasks valid	> Chance face gaze	> Chance face gaze	> Chance face gaze	> Chance biomotion gaze	Pupil constriction post flash
Null hypothesis	–	%Face = 3.2%	%Face = 8.3%	%Face = 3.9%	%Bio = 50%	Constrict > 0
Sample values	AM✓ SI✓ SS✓	*M* = 27.6%, SD = 8.5%	*M* = 30.4%, SD = 9.6%	*M* = 34.9%, SD = 8.0%	*M* = 54.8%, SD = 6.1%	*M* = .505, SD = .074
Statistic	–	t(118) = 31.3	t(118) = 25.0	t(118) = 42.1	t(118) = 8.5	t(116) = 73.9
*p*	–	< .001	< .001	< .001	< .001	< .001
*d*	–	2.87	2.30	3.88	.79	6.83
Validity	✓	✓	✓	✓	✓	✓

Group	*df* range	OMI	AM %Face	SI %Face	SS %Face	BM %Bio	PLR latency
*Six-week stability (ICC) from T1 to T2 for primary pre-specified biomarkers*							
TD	106 ≤ *df* ≤ 112	.828	.848	.842	.569	.441	.866
ASD	223 ≤ *df* ≤ 274	.836	.834	.813	.680	.505	.749
ASD < 8.5 years	116 ≤ *df* ≤ 144	.837	.820	.813	.657	.462	.775
ASD ≥ 8.5 years	106 ≤ *df* ≤ 129	.832	.842	.812	.695	.530	.725
ASD IQ < 75	24 ≤ *df* ≤ 30	.754	.801	.794	.476	.238	.741
ASD IQ ≥ 75	198 ≤ *df* ≤ 243	.849	.836	.819	.701	.526	.749

	OMI	AM %Face	SI %Face	SS %Face	BM %Bio	PLR latency
*Group discrimination at T1 for primary biomarkers*						
M (SD)						
ASD values	24.4% (8.5%)	18.7% (8.7%)	24.2% (10.7%)	29.9% (9.8%)	53.4% (6.8%)	285 ms (15 ms)
TD Values	30.9% (7.6%)	27.6% (8.5%)	30.4% (9.6%)	34.9% (8.0%)	54.8% (6.1%)	279 ms (15 ms)
No covariates						
Statistic	F(1,389) = 55.6	F(1,397) = 90.7	F(1,393) = 31.5	F(1,390) = 27.7	F(1,394) = 4.0	F(1,381) = 10.1
*p*	< .001	< .001	< .001	< .001	.046	.002
*d*	*− *.788	*− *1.037	*− *.593	*− *.537	*− *.211	.350
*ηp*^*2*^	.117	.184	.069	.058	.009	.025
Age + IQ + Acq + Site Control						
Statistic	F(1,382) = 24.5	F(1,390) = 55.3	F(1,386) = 21.8	F(1,383) = 3.9	F(1,387) = 1.9	F(1,374) = 6.7
*p*	< .001	< .001	< .001	.048	.165	.010
*ηp*^*2*^	.057	.120	.050	.009	.006	.018

Phenotypic characteristic	OMI	AM %Face	SI %Face	SS %Face	BM %Bio	PLR Latency
*Spearman’s Correlations between ET and child behaviors in the ASD group at T1*						
Age	*.115*	.147*	*.077*	*.087*	*− .063*	.153*
Full IQ	.123*	.140*	*.033*	.192**	*− .063*	*− .006*
Verbal IQ	.183**	.188**	.122*	.199***	*− .058*	*.026*
NV IQ	*.059*	*.081*	*− .029*	.147*	*− .072*	*− .030*
ADOS SA	*− *.165**	*− *.228***	*− *.110	*− *.168**	.041	*− *.018
ADOS RRB	*− *.035	*− *.087	.001	.004	.024	*− *.100
VABS3 Soc SS	.125*	.150*	.103	.118	.008	*− *.032
VABS3 Com SS	.182**	.205***	.144*	.203***	.029	*− *.049
SRS SCI T	*− *.070	*− *.059	*− *.090	*− *.062	*− *.117	.096
SRS RIRB T	*− *.108	*− *.090	*− *.119*	*− *.092	*− *.041	.086
PDDBI SocApp T	.066	.130*	.079	.038	.146*	*− *.058
PDDBI REPRIT T	*− *.238***	*− *.211***	*− *.210***	*− *.191**	*− *.001	.106
Face Memory SS	.316***	.359***	.256***	.301***	*− *.045	.051

OMI —Oculomotor Index of Gaze to Human Faces; BM = Biological Motion Preference; PLR — Pupillary Light Reflex; AM = Activity Monitoring; SI—Social Interactive; SS = Static Scenes; **[Construct Validity]** Task —ET task; Construct —hypothesized construct under investigation; Test —how the construct is tested; Null Hypothesis —formal definition of the construct validity test; Sample Values —TD performance on null hypothesis variable at T1; **[Signal Acquisition]** Acquisition Valid —participant generated ET data for some portion of the assay; Signal Valid —valid signal for primary DV (meeting all quality control criteria for admission of data); Difference in Site Valid Signal Rates —Pearson’s Chi-Squared test for site differences in valid signal (consistent with Monte Carlo simulation and unable to be computed for 100% data validity); **[Six-week Stability]** ICC —Intraclass Correlation Coefficients (ICC3); DV — dependent variable; IQ —DAS Full Scale IQ; df — degrees of freedom across task DVs in calculation of ICCs; **[Group Discrimination]** DV —dependent variable; Age = participant age; IQ = Full Scale IQ; Acq = %Valid data collection rate; Site = data collection site; *d* = Cohen’s d; *ηp2* = partial eta squared; **[Phenotypic Characteristic Correlations]** Full IQ = DAS Full Scale IQ; NV IQ = DAS Nonverbal IQ; ADOS SA — Autism Diagnostic Observation Schedule social affect comparison score; ADOS RRB —restricted interests and repetitive behavior comparison score; VABS3 Soc — Vineland Adaptive Behavior Scales adaptive behavior socialization standard score; VABS3 Com — communication standard score; SRS-2 SCI —Social Responsiveness Scale social communication and interaction T-score; SRS-2 RRB — restricted interest and repetitive behavior T-score; PDDBI SocApp T — Pervasive Developmental Disorders Behavior Inventory Social Approach Behaviors T-score; PDDBI REPRIT — Repetitive, Ritualistic, and Pragmatic Problems Composite T-score; Face Mem SS — NEPSY memory for faces subtask score. ^*^p < .05; ^**^p < .01; ^***^p < .001. Underlined cells are significant even after controlling for Age, Full Scale IQ, and %Valid Data. *Italicized* cells cannot be controlled for these variables due to collinearity

### Construct validity

To ascertain whether tasks successfully tapped constructs of interest, we examined pre-defined hypotheses for each task in the TD group (Tables 2, S11a). These hypotheses primarily served to verify that tasks were eliciting expected responses from TD children based on their intended design. ActivityMonitoring, SocialInteractive, and StaticScenes tasks were all designed wholly or in part to examine attentional predispositions for directing gaze toward social information as present in faces, motivated by studies indicating that faces are a privileged target for visual attention in TD individuals [36, 37]. For these tasks, we used one-sample t-tests of %Face against the scene percentage occupied by the Face region, examining whether completely randomly directed attention could explain the proportion of time spent by TD children looking at faces. As a stronger benchmark, we also used a variation of the most well-studied low-level computational model of visual saliency [38], extended for motion saliency calculation [39, 40], to compute gaze probability fields (see Supplemental Material for additional notes on Construct Validity). For Biomotion, construct validity tested biological motion preference, i.e., greater than chance looking at biological compared to control motion (one-sample t-test against 50%), reflecting attentional preferences for biological movements as expected in typically developing individuals [41, 42]. For PLR, we tested whether the pupil constricted after the screen flash (one-sample t-test against 0), indicating expected behavior of the pupil to light [43].

### Six-week stability

In the ASD and TD groups, we assessed short-term stability of individual biomarkers from T1 to T2 (~ 6 weeks) using intraclass correlation (ICC, via two-way mixed models with absolute agreement) (Table 2). We defined ICC ≥ 0.5 as a moderate relationship and ICC ≥ 0.75 as a high relationship across 6 weeks. To examine whether participant age or IQ influenced stability within the ASD group, we also examined children younger and older than 8.5 years of age and with IQs below or above 75. We distinguish six-week stability from a focus on test–retest reliability, which would require repetition of the biomarker assessments in close temporal proximity on the scale of hours or days.

### Group discrimination

We examined group discrimination at T1 and T2 using ANOVAs (Tables 2, S12ab) with heteroskedasticity consistent covariance matrix (HC3) correction due to unequal group variances. To verify that results were not driven by age, IQ, site, or %Valid Data, we included them as simultaneous covariates in follow-up models. We note that the development of a discrimination biomarker is not the primary intention of this analysis. Rather, examination of between-group discrimination serves two purposes. First, because biomarkers were selected on the basis of prior findings and preliminary studies, it is necessary to replicate prior findings so as to verify the reproducibility and generalizability of targeted constructs. Because the foundational literature associated with ET paradigms all involve between-group differences in biomarker performance, this process served as a “secondary construct validity criteria,” providing evidence that ET biomarkers were performing “as expected.” Second, because the selected ET biomarkers were developed to investigate mechanistic phenomena, the presence of between-group differences (especially in reference to a typically-developing control population) signifies atypical function of associated mechanisms at a group level in ASD. These differences are not expected to have effect sizes at the level of individual diagnostic precision, but rather to associate with broad group-level distributional asymmetries in biomarker performance. These asymmetries, in turn, are expected to point to the presence of more homogeneous subsets within the heterogeneity of the autism spectrum, allowing for the indexing of individuals within the autism spectrum with specific patterns of outlying biomarker performance.

### Clinical correlations

To examine the extent biomarkers could explain known heterogeneity and areas of vulnerability in ASD, we examined relationships between biomarkers and clinical and behavioral characteristics at T1 in the ASD group (Tables 2, S13a). As with acquisition measure correlations with clinical phenotype, analyses were conducted using Spearman’s correlations both with and without partialing for age, IQ, and %Valid Data (with comparisons of Pearson’s and Kendall’s correlation in Additional file 1: Tables S13a1 and S13a2, respectively).

## Results

### Acquisition

As shown in Table 2 and Additional file 1: Table S6a, acquisition and valid signal rates at T1 for all derived variables were high (> 95%). Signal validity differed across sites only for the ASD group in the PLR task, but overall data loss in this task was low (*n* = 14 invalid out of 280 children with ASD) suggesting minimal impact on overall study metrics. At T2, PLR signal validity was lower, but other tasks continued showing high performance (> 95%) (Additional file 1: Table S6b).

The ASD group provided less high-quality data (i.e., lower percentage of valid trials, lower valid data per valid trial, and worse calibration error) than the TD group (Additional file 1: Table S7). After controlling for age, IQ, and site differences, group difference effect sizes diminished across acquisition metrics. In the ASD group, lower data quality was broadly associated with lower cognitive ability and greater ASD-related symptoms (Additional file 1: Table S8). Lower quality of acquisition metrics in ASD were also associated with lower values of ET biomarkers indexing gaze to people and faces (but not PLR or Biomotion variables, Additional file 1: Table S9), as well as lower quality with other acquisition metrics (Additional file 1: Table S10).

### Construct validity

All tasks induced above-chance performance in the TD group (Tables 2, S11a). Use of a saliency map baseline for %Face evaluation of ActivityMonitoring, SocialInteractive, and StaticScenes did not affect overall result patterns, though effect sizes diminished (see Supplemental Information discussion on Construct Validity). Effects for Biomotion, while significant, were modest compared to other tasks. Similar results were found in ASD (Additional file 1: Table S11b).

### Six-week stability

**Fig. 2:**
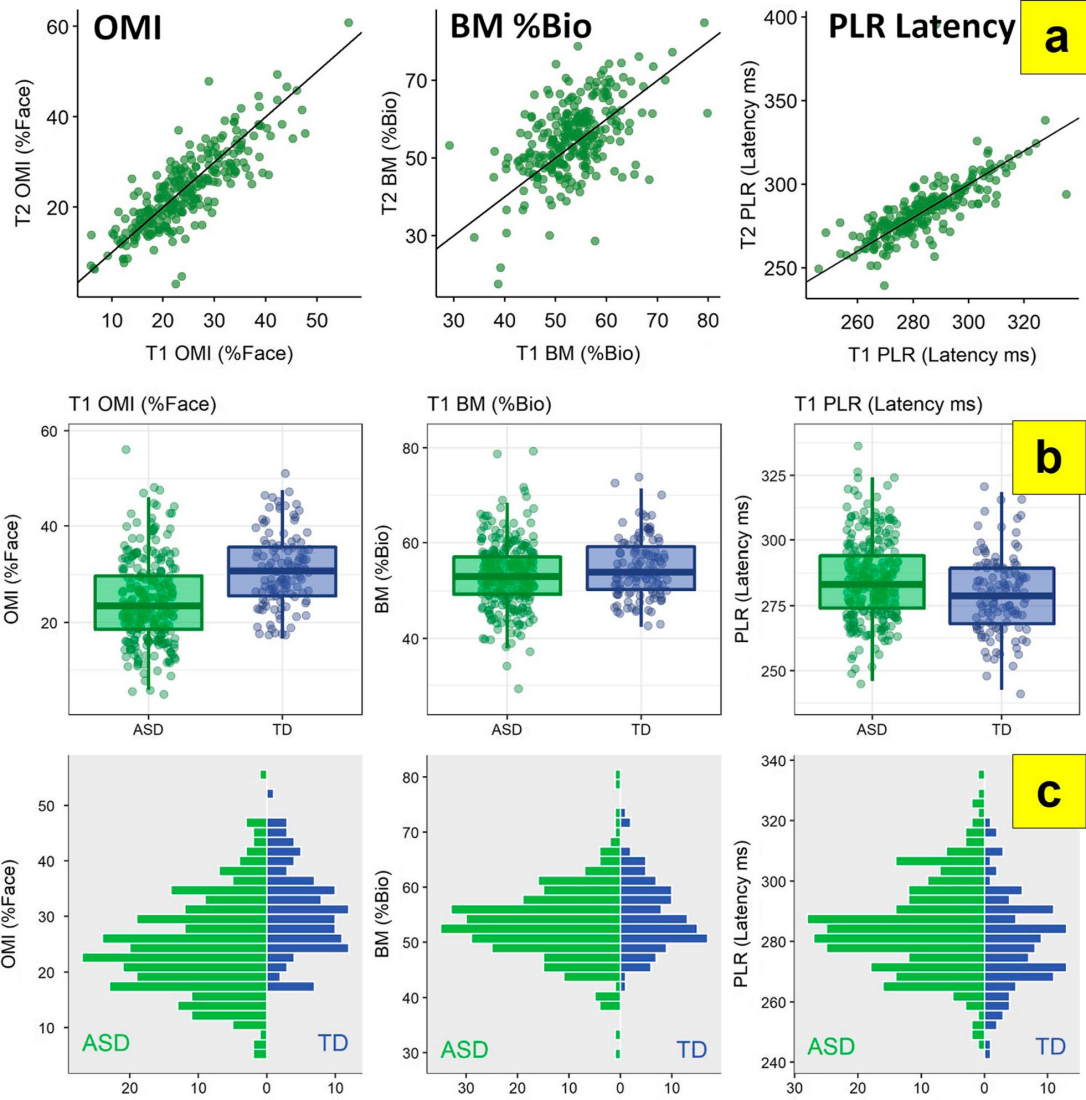
**A** Six-week stability (T1 to T2) in the ASD group; **B** T1 ASD vs. TD boxplots; and **C** T1 ASD versus TD histograms for Oculomotor Index of Gaze to Human Faces (OMI), Biomotion (BM) %Bio, and PLR Latency Biomarkers. Diagonal line in stability charts is identity (slope = 1). See Supplemental Figures S2-S3 for additional biomarkers

All variables exhibited moderate (≥ 0.5) or high (≥ 0.75) ICCs in both ASD and TD groups except for SocialInteractive %Social and Biomotion %BioAffect (both groups) and Biomotion %Bio (TD group) (Table 2; Figs. 2a, S2). In the ASD group, this pattern was preserved for children ≥ 8.5 years and IQs ≥ 75. ICCs for Biomotion %Bio were low for children < 8.5 years of age. Biomotion %Bio, StaticScenes %Face, and ActivityMonitoring %Activity were low for children < 75 IQ.

### Group discrimination

All primary measures showed between-group differences (Tables 2, S12a; Figs. 2b, S3). Compared with TD children, children with ASD had lower OMI scores, looked less at faces in ActivityMonitoring, SocialInteractive, and StaticScenes tasks, looked less at biological motion, and had later PLR latencies. Only Biomotion differences became non-significant when controlling for age, IQ, site, and %Valid Data. Effect sizes for %Face variables and the OMI ranged from moderate (StaticScenes: *d* = 0.537) to large (ActivityMonitoring: *d* = 1.037).

There were no significant differences with or without covariate adjustment for most secondary variables including looking at activities (ActivityMonitoring), looking at biological motion during affective trials (Biomotion), and relative pupil constriction (PLR). Between-group differences in looking at social information were significant in SocialInteractive with or without adjustment, and for StaticScenes only without adjustment. T2 between-group differences were numerically similar to results at T1 (Additional file 1: Table S12b) with the exception of PLR latency, which was comparable in ASD and TD participants at T2 with or without adjustment.

### Clinical correlations

**Fig. 3:**
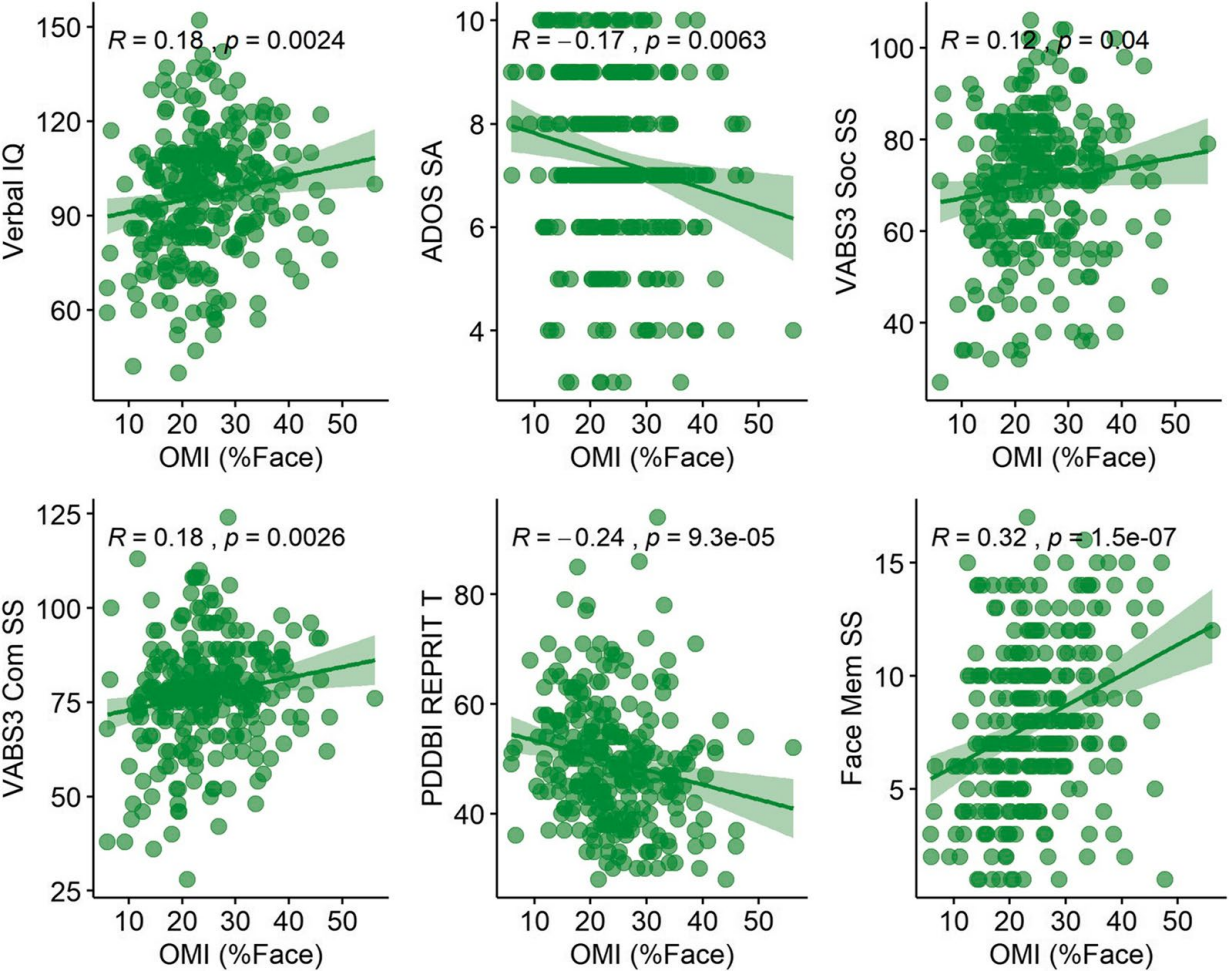
Oculomotor Index of Gaze to Human Faces (OMI) relationships with child characteristics in the ASD group at T1. Spearman’s Correlation Coefficient and *p* value reported

Correlations are shown in Table 2 (select relationships with OMI, Fig. 3). In the ASD group, diminished looking at faces (OMI, ActivityMonitoring, SocialInteractive, and StaticScenes) was associated with greater presence of autism-related symtpoms as measured by ADOS Social Affect Comparison Scores, VABS3 Communication Standard Scores, and the PDDBI Repetitive, Ritualistic, and Pragmatic Problems Composite (REPRIT/C) Scale, as well as with worse NEPSY Memory for Faces scores. Overall gaze toward human figures in SocialInteractive and StaticScenes tasks showed similar associations. When age, IQ, and %Valid Data were controlled, relationships between looking at faces and VABS3 Communication, PDDBI REPRIT/C, and NEPSY Memory for Faces remained significant; by contrast, ADOS Social Affect became significantly associated only with ActivityMonitoring %Face.

In the TD group, associations were similar, with more notable relationships with age (Additional file 1: Table S13b).

## Discussion

This study evaluated candidate ET biomarkers by testing pre-specified primary and secondary variables from five assays. We examined key biomarker attributes relevant to their use in clinical trials, including valid acquisition rates, construct validity, short-term six-week stability, group discrimination, and association with clinical measures.

### Acquisition

Valid acquisition rates at T1 were high (> 95%) for both ASD and TD groups, surpassing our predefined adequacy criterion (> 70%). Site differences were not observed for ROI-based biomarkers, supporting their acquisition robustness. However, site differences were observed for PLR in the ASD group. While the data loss rate was low, further scrutiny of PLR tasks in regard to interactions with individual characteristics or environmental variation (e.g., lighting conditions) is warranted.

Generally, across metrics and tasks, the ASD group showed lower data quality than the TD group. This was expected, as multiple studies have shown that children with ASD and other developmental conditions have lower levels of with compliance and attention during experimental tasks (e.g., see [9, 44]). In the ASD group, lower data quality was associated with more pronounced differences compared to the TD group on a range of clinical measures, including IQ and social abilities. Relationships with autism symptoms remained significant even when controlling for age and IQ. It is important to note, however, that good data quality was found in both ASD and TD groups: averaging across experiments, ET data were acquired for 87.1% of trials on average in the ASD group (94.0% in the TD group); calibration error averaged 0.607˚ (TD: 0.534˚); and 90.9% of trials were valid overall (TD: 93.0%). These findings reinforce the feasibility of ET data acquisition in ASD as well as the relatively nuanced relationships between data quality and clinical features.

It is important to note that most experiments contained information of a social nature. Given the primacy of differences in social behavior and attention in children with ASD [8], it is possible that lower rates of data acquisition (including inattention to stimuli) share mechanistic relationships with diminished social information seeking. That is, children with the most pronounced differences in social abilities compared to TD were the most inattentive to social stimuli overall during the task, resulting in increased data loss. This is supported by significant relationships identified between acquisition metrics and ET face-focused biomarkers and the lack of relationships observed between acquisition metrics and general biological motion and pupillary light reflex tasks.

It should be noted that data quality measures, by themselves, lack specificity for ASD and could be associated with a wide range of psychiatric and clinical conditions including ADHD and cognitive impairment. In contrast, diminished looking at faces is consistently evident in individuals with ASD as compared to developmental- and chronological-age-matched controls. In this study, diminished face looking was computed as a proportion of validly collected data, theoretically conditioning it against the effects of general data loss. However, exposure to the social information present in faces scales by both the proportion of time spent looking at faces as well as the total time spent looking at the scene. Future work should explore the nature of relationships among data quality, ET biomarkers, and clinical characteristics, as well as the mechanisms and significance underlying poorer acquisition metrics (i.e., calibration error, lost data, and lost trials) in ASD.

### Construct validity

Pre-specified criteria for construct validity, as measured in the TD group, were robustly demonstrated for PLR, OMI, and OMI-associated tasks. Biological motion preference, while meeting expectations, exhibited effect sizes 3–5 × smaller than face preference tests, and 9 × smaller than pupil constriction, suggesting the construct it assays may not be as robust as other biomarkers. While construct validity was only expected to be verified in the TD group, results in ASD were similar, suggesting applicability to ASD as well.

### Six-week stability

We focused on stability between baseline and the six-week timepoint to parallel a short-term clinical trial. Measures indexing attention to faces in dynamic scenes (OMI, ActivityMonitoring, and SocialInteractive) and PLR measures showed strong stability in both ASD and TD groups. These results, combined with their relatively invariant performance in ASD subgroups based on age or IQ, demonstrate promising viability of these biomarkers for indexing stable characteristics of children over time.

Several measures had lower ICCs for six-week stability, potentially for different reasons. Gaze toward social information (bodies, heads, and activities) in the Social Interactive Task may have had ceiling effects (TD: 91.6%, ASD 85.8%). Relative instability of biological motion preference in trials depicting affective content may have been due to a reduced trial count (20% of Biomotion trials). However, it is also possible measures with lower stability index state-like participant attributes, whereas biomarkers with higher stabilities index trait attributes.

### Group discrimination

All primary variables showed expected ASD-TD differences. Between-group differences were especially prominent for the OMI, gaze toward faces in the Activity Monitoring Task, and gaze at general social information in the Social Interactive Task. Controlling for variability in age, IQ, site, and quantity of valid data did not change the significance pattern of most variables, suggesting these variables may reflect intrinsic differences in social-attentional processing between groups.

Group differences in biological motion preference, however, became non-significant after covariate adjustment. This suggests that the Biological Motion Preference Task may not be as robust in terms of between-group differences as other tasks. Similarly, PLR latency may be more variable than other biomarkers in terms of group discrimination as indicated by a loss of significance from baseline to six-week follow-up.

### Clinical correlations

Multiple relationships between biomarker variables and clinical measures were found. Decreased gaze toward faces was associated with the presence of greater differences in social performance, both when measured behaviorally and by parent report. Relatedly, it was associated with verbal IQ but not nonverbal IQ, suggesting stronger associations with communicative competence rather than general cognitive ability. Of note, the strongest relationship of gaze to faces was found with memory for faces, suggesting shared mechanisms between looking at faces and ability to remember them. These results support relationships between gaze toward faces and social communicative function.

However, the strength of biomarker-clinical relationships were in general small, moving into the medium effect-size range [45] only for memory for faces. These moderate relationships are of varying import depending on application. For direct prediction of outcome measures or use as surrogate endpoints of a measure, strong associations may be most critical. For other applications, e.g., in the case of stratification of samples, relationships with clinical variables may be less critical. The modest relationships observed suggest potential utility for applications such as these.

### Biomarker viability

We evaluated our proposed tasks and task variables on multiple properties (acquisition, construct validity, six-week stability, group discrimination, and clinical relationships) relevant to their potential as a biomarker for use in clinical trials for children with ASD. PLR variables showed good six-week stability but did not show stable group differences over two timepoints or correlations with child characteristics. Biological motion preference tasks showed suboptimal six-week stability, weaker group discrimination, and few associations with child characteristics.

Gaze toward faces, across multiple tasks and assays, fully met expectations on all evaluated criteria. Based on these results, and in consideration of its associations with socio-communicative ability as well as its history in literature as a strong discriminator between ASD and controls [13], a Letter of Intent for the OMI biomarker (“Oculomotor Index of Gaze to Human Faces”) was submitted and subsequently accepted to the FDA’s Center for Drug Evaluation and Research Biomarker Qualification Program.

While many biomarkers presented here perform adequately across multiple dimensions, and though large between-group effects were observed on a number of variables indexing social attention, considerable distributional overlap exists between ASD and TD groups on all measures. For this reason, none of the ET biomarkers would be viable as a biomarker to identify categorical diagnosis. Evidence from this study suggests a more appropriate context of use may be stratification, or the identification of subgroups within the autism spectrum that are more homogeneous in terms of their social-attentional profiles (and potential underlying biology). From this perspective, between-group analyses reflect distributional differences associated with a sizeable number of children with ASD in the “tail” of the TD OMI distribution, pointing toward a potential subgroup within the autism spectrum unified by diminished gaze to faces. Conversely, it also provides information about children with ASD with more typical levels of gaze to faces – specifically, that the nature of their autistic symptoms may be less likely to be associated with atypical visual social cognitive strategies. It is important to note, however, that both interpretations (and the use of the TD population, in general, to define an expected “normative” range of biomarker function) are subject to, among other issues, diagnostic imprecision and biases associated with categorical delineations. Alternative approaches could consider continuum-based interpretations of ET biomarker heterogeneity (and relationships of that heterogeneity to other performance domains) from a population perspective.

Associations between ET biomarkers and behavioral characteristics were generally small in effect size. This suggests that ET biomarkers would be unlikely to serve as a direct proxy for the clinical measures examined. However, their overall consistency and patterns of significant relationships suggests that they may capture variance associated with clinically meaningful heterogeneity in ASD. A key question is how ET biomarkers, as compared to more traditional clinical variables, may serve in the landscape of clinical trials for ASD. While the OMI and associated ET variables lack strong associations with clinical symptoms of ASD, they provide precision in the measurement of mechanistic constructs related to spontaneous orienting and sustained attentional engagement with socially-relevant visual scene characteristics. The goal of biomarker research in ASD is not necessarily to recapitulate or reproduce variation already well-established or well-represented by extant clinical measures. Indeed, the notion that a distal, mechanistic marker would provide greater or even equal accuracy in the measurement of clinical, behavioral symptoms of ASD than direct measures of those clinical, behavioral symptoms, seems unlikely and of questionable utility. Rather, the establishment that a given biomarker is practically viable in terms of key psychometrics leads naturally to a subsequent goal: the identification, evaluation, and validation of downstream applications and specific contexts-of-use focused upon the biomarker constructs.

The OMI has the potential to aid in the stratification of a more homogeneous subgroup within the heterogeneity of the autism spectrum. Clinical trial applications related to this context of use include predictive biomarkers to stratify likely responders to specific interventions (e.g., interventions focused on improving motivation to look at faces would likely be more successful in low-OMI participants; interventions focused on improved decoding of emotional and non-verbal face cues would likely be more successful for high-OMI participants); prognostic biomarkers aiming to predict likely concurrent or later emerging vulnerabilities in specific domains (e.g., missing nonverbal cues in conversational turn-taking in individuals with low-OMI); and response biomarkers of therapies expected to impact social motivation for (and consequently, attentional biases to) faces.

### Limitations

As an observational study with no strict interventional prescription, however, this work offers limited information regarding how the selected ET biomarkers track or predict outcomes in response to specific interventions. Further studies will be needed to evaluate the potential of these ET biomarkers to serve in different contexts-of-use.

Investigation of the possible impact of psychiatric conditions highly comorbid with ASD such as ADHD, anxiety, and mood disorder [46, 47] would further our understanding of the ability of ET biomarkers to disentangle subset populations within a clinical setting. For example, prior work has shown that children with combined ASD and ADHD, unlike children with ASD without ADHD, show reduced fixation duration to faces when looking at low-complexity static social stimuli compared to TD children [48]. While our analyses were structured to mitigate confounds due to diminished overall task attention—a trait that might be expected to be common along multiple psychiatric axes, including ADHD—further investigation is merited. Additionally, examinations of sex/gender effects should be more formally evaluated by future work. For example, prior work has highlighted attentional sex differences in children with ASD on the Social Interactive ET paradigm [49]. While we include preliminary analyses in Supplemental Information suggesting that study findings are unlikely to be strongly impacted by sex differences, the question of sex effects on ET biomarkers involves many more nuances than have been considered in this current report. It is highly likely that in-depth exploration of these two characteristics of psychiatric comorbidities and sex differences will improve the precision of future biomarker applications, increase our appreciation of heterogeneity in ASD, and potentially lead to new clinical insights.

From a methodological standpoint, while eye tracking technologies have become affordable (e.g., see [50]), this current study was conducted on more costly research-grade high-performance systems. Future work should consider the tradeoffs and sufficiency of lower-cost eye-tracking systems as platforms for ET biomarker acquisition. In addition, while studies of social-attentional constructs are preponderant in ASD research, the ET battery presented in this study represents only a fraction of constructs that may be indexed using ET technologies. Our use of the term social attention is intended to refer operationally to visual attention to social content within a stimulus and does not incorporate the full range of potential applications of this term. And while a broad social-attention-focused approach is sensible and appropriate for this first generation of ET biomarker development for ASD, subsequent refinements and iterations may be required to isolate mechanistic targets informing therapeutics. Similarly, the conceptualization of social attention itself is an area of active exploration [51], encompassing a wide range of phenomena from fast-acting and dedicated brain circuitry involved in processing of faces and socially-relevant nonverbal cues [52, 53] to context-integrative systems impacting attentional bias for peers due to social status [54], personal significance [55], and emotionality [56–58]. The ET biomarkers examined in this study index only a small slice of possible social-attentional constructs, most prominently the spontaneous orienting and sustainment of gaze to human faces in viewing contexts of interactive and solitary human activities. The social nature of this “face looking” construct rests on assumptions regarding reciprocal relationships between looking at faces and social motivation, perception, cognition, and behavior. While supported by identified relationships between ET biomarkers and ostensibly social functions such as social-affective behaviors, communication skills, and memory for faces, alternative interpretations of ET biomarkers such as the OMI should be considered. These alternatives include cognitive models that might consider limited attention toward faces as reflections of more generally atypical information processing strategies [59–61]. Through such a diversity of such views, the multiple convergent pathways by which a low or high-OMI could be achieved could itself be decomposed, and in doing so achieve even greater precision in characterizing individual variation and robustness in deconstructing group heterogeneity.

This study similarly suggests further optimization of ET biomarkers may be possible. For example, we note that stability properties of percentage of looking at faces in the static scene task was lower than that of activity monitoring and social interactive tasks. Similarly, the activity monitoring task and social interactive task were individually comparable in performance to the overall OMI. Reweighting, or exclusion, of measures comprising the OMI may improve its overall psychometric properties, with a logical first step being a focus on the “best content” from each task rather than exclusion of tasks in their entirety. In addition, the current ET battery is conducted over two days. Reducing the battery to a single session of minimal duration will yield large benefits for practical deployment in clinical trials. Ongoing work aims to identify thresholds for stratification, improve psychometric properties through variable refinement (e.g., by reinspection of subtasks contributing to OMI performance), optimize tradeoffs between performance and usability, investigate mechanistic relationships between data quality and ET variables, and explore application areas. Toward these purposes, it is our hope that this study provides important initial baseline information for the development and evaluation of extant and future ET biomarkers for ASD.

## Conclusions

Our results suggest the examined ET measures, especially gaze to human faces, show good properties in terms of common requirements for biomarker applications in clinical trials including: feasibility in valid data acquisition, verification of construct performance, stability over six-weeks, between-group differences consistent with prior literature and indicative of atypical performance in subsets of children with ASD, and associations with clinical measures. Further work is necessary to develop and validate examined measures in specific biomarker applications.

## Supplementary Information

**Additional file 1**. Additional details, including information on methods and materials (study protocol, participant characteristics, data acquisition, experimental tasks, and analytical plan), results (acquisition, construct validity, six-week stability, group discrimination, clinical correlations, and preliminary analyses of sex effects), and manual references.

## Data Availability

Preliminary data were reported at the International Society for Autism Research 2017–2020 (https://www.autism-insar.org/page/MeetingArchives). Protocols and manuals are available at https://medicine.yale.edu/ycci/programsprojects/autism/postersandpapers/. The project is listed in ClinicalTrials.Gov NCT02996669. Repository Data are available from NIMH NDA (#2288) (https://nda.nih.gov/edit_collection.html?id=2288).

## Footnotes

1 Though no between-group differences were noted in sex and the current study was not powered for a rigorous investigation of sex-effects in ASD, consideration of sex differences on biomarker performance remains an important topic. We consider this in a set of preliminary analyses at the end of Supplemental Information. These analyses suggest that biological sex may have limited impact on the overall patterns of results highlighted in this report.
