# Supplementary material for: The Autism Biomarkers Consortium for Clinical Trials: evaluation of a battery of candidate eye-tracking biomarkers for use in autism clinical trials
Source: Mol Autism. 2022 Mar 21;13:15. doi: 10.1186/s13229-021-00482-2 (PMC10124777; doi:10.1186/s13229-021-00482-2)
Supplement: Supplementary file 1 — Additional file 1. Additional details, including information on methods and materials (study protocol, participant characteristics, data acquisition, experimental tasks, and analytical plan), results (acquisition, construct validity, six-week stability, group discrimination, clinical correlations, and preliminary analyses of sex effects), and manual references. [file 13229_2021_482_MOESM1_ESM.docx]

# Supplemental Information

# Methods and Materials

## Autism Biomarker Consortium for Clinical Trials (ABC-CT) Protocol

The Autism Biomarkers Consortium for Clinical Trials (ABC-CT) study was a observational, longitudinal study focused on characterizing and validating experimental measures (including eye tracking (ET) and electroencephalography (EEG) experiments) for use as biomarkers in clinical trials for children with autism spectrum disorder (ASD). The ABC-CT consisted of five implementation Sites: Boston Children’s Hospital, Duke University, UCLA, University of Washington, and Yale University. These Sites independently recruited families of children with ASD and children with typical development (TD) to participate in a three-time-point longitudinal study involving caregiver surveys and interviews, and direct behavioral assessments and experimental batteries delivered to children.

Children with ASD or TD participated in the first visit of three timepoints (Time 1/T1/Baseline) between the ages of 6 years 0 months to 11 years 6 months. Subsequent timepoints were targeted for 6 weeks (±2 weeks) after baseline (Time 2/T2) and 6 months (±2 weeks) after baseline (Time 3/T3). This report examines only information regarding T1 and T2. Each visit/timepoint included two ET sessions which occurred on two different days, with the second day targeted for 1 to 14 days subsequent to the first day. Actual days between T1 ET sessions were *M*=6.01 days (*SD*=4.30, range 1-22 days) for the ASD group, and *M*=6.30 (*SD*=4.55, range 1-14 days) for the TD group, with no statistical differences observed between groups (*t*(207.0)=.584, *p*=.560). Actual days between T2 ET sessions were *M*=6.21 days (*SD*=5.14, range 1-41 days) for the ASD group, and *M*=6.92 (*SD*=4.50, range 1-21 days) for the TD group, with no statistical differences observed between groups (*t*(237.4)=1.362, *p*=.175). The first day of T2 occurred after the first day of T1 *M*=5.94 weeks (*SD*=1.34, range 3.71-8.29 weeks) in the ASD group, and *M*=5.85 weeks (*SD*=1.10, range=4-8.14 weeks) in the TD group, with no significant between-group difference (*t*(251.0)=.707, *p*=.480). Participants were not removed from the study or subsequent analyses for falling outside ideal dates on either a session (day-to-day within a timepoint) or on a timepoint-to-timepoint basis. However, one TD participant withdrew from the study before T1 Day 2; five TD and four ASD participants withdrew before T2 Day 2.

## Participant Characteristics

Sample characteristics are provided in **Table 1** for the total sample at T1. Given that all participants (both in the TD group and the ASD group) provided some usable eye-tracking data at T1, the sample characteristics of the total sample are identical to the sample characteristics of those participants providing valid eye-tracking data. Given high rates of valid experimental data acquisition, as highlighted in **Table 2 and Table S6ab**, sample characteristics considering only children with valid data on an experiment-by-experiment basis varied little from this overall profile (**Tables S1ab**).

#### **Table S1a. Characteristics for Participants with Valid Data for OMI, Biomotion, PLR ET Biomarkers at Time 1.**

Mean and standard deviation are presented for clnical assessments for individuals included within each experimental analysis at baseline.
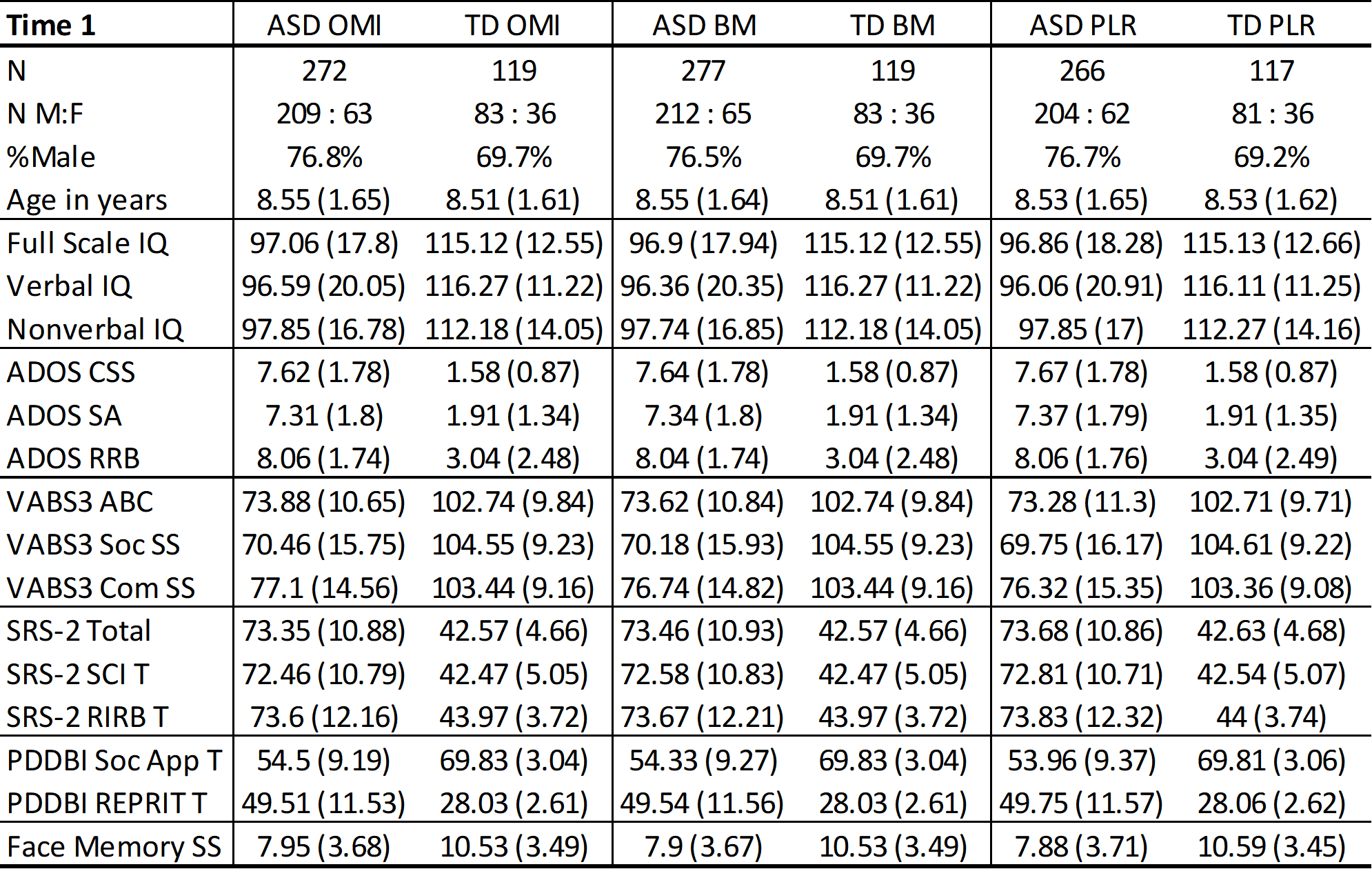


Table Key: OMI=Oculomotor Index of Gaze to Human Faces; BM=Biological Motion Preference Task; PLR=Pupillary Light Reflex Task; N M:F = number of males:females; ADOS CSS = Autism Diagnostic Observation Schedule calibrated severity score (comparison score); ADOS SA = social affect comparison score; ADOS RRB = restricted interests and repetitive behavior comparison score; VABS3 ABC = Vineland Adaptive Behavior Scales adaptive behavior composite standard score; VABS3 Soc SS = socialization standard score; VABS3 Com SS = communication standard score; SRS-2 Total = Social Responsiveness Scale total T-score; SRS-2 SCI T = social communication and interaction T-score; SRS-2 RIRB T= restricted interest and repetitive behavior T-score; PDDBI Soc App T = Pervasive Developmental Disorders Behavior Inventory Social Approach Behaviors T-score; PDDBI REPRIT T = Repetitive, Ritualistic, and Pragmatic Problems Composite T-score; Face Memory SS = NEPSY memory for faces subtask score.

#### **Table S1b. Characteristics for Participants with Valid Data for ActivityMonitoring, SocialInteractive, StaticScenes ET Biomarkers at Time 1.** Mean and standard deviation are presented for clinical assessments for individuals included within each experimental analysis at baseline.


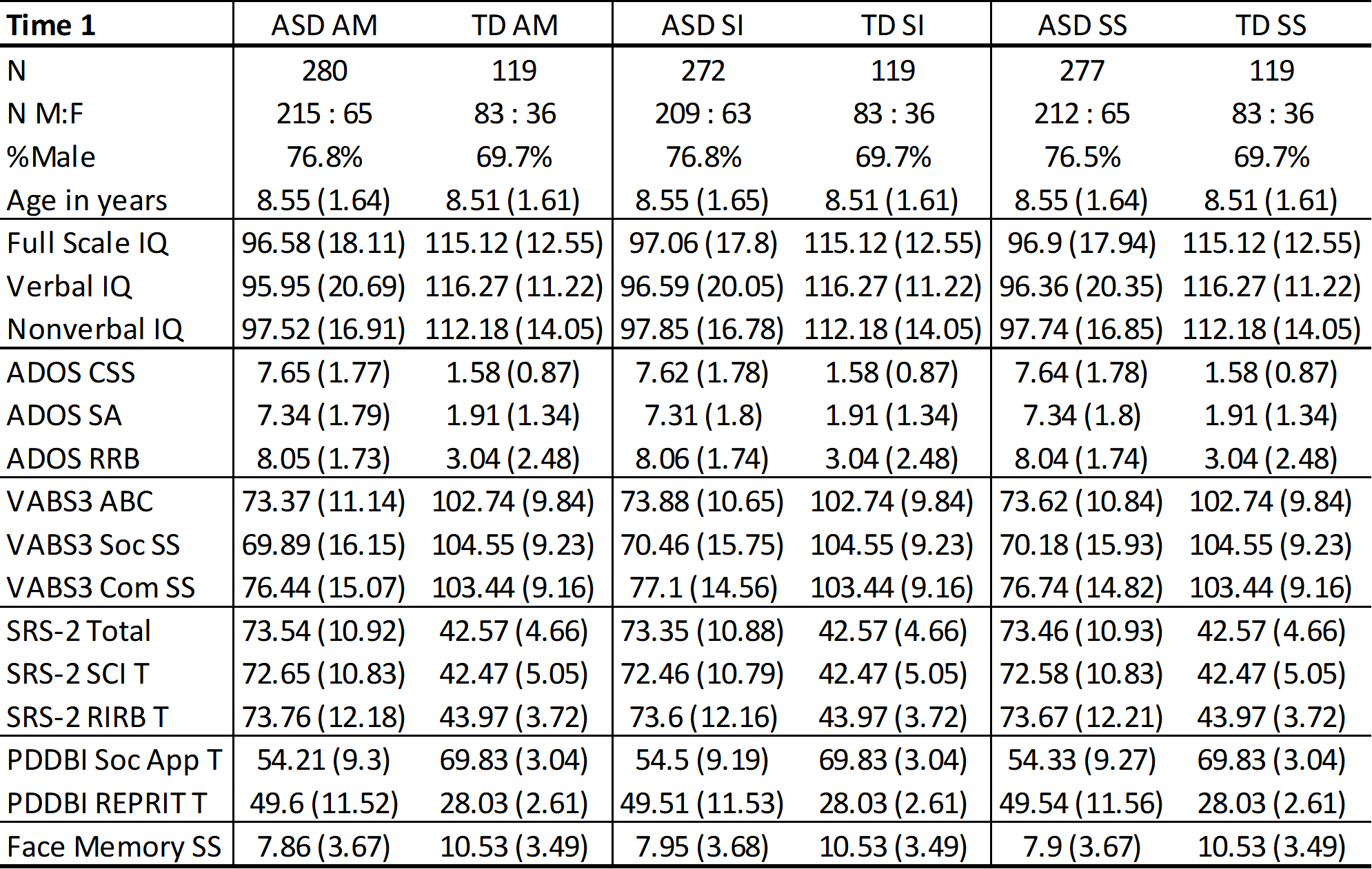


Table Key: AM=Activity Monitoring Task; SI=Social Interactive Task; SS=Static Social Scenes Task; N M:F = number of males:females; ADOS CSS = Autism Diagnostic Observation Schedule calibrated severity score (comparison score); ADOS SA = social affect comparison score; ADOS RRB = restricted interests and repetitive behavior comparison score; VABS3 ABC = Vineland Adaptive Behavior Scales adaptive behavior composite standard score; VABS3 Soc SS = socialization standard score; VABS3 Com SS = communication standard score; SRS-2 Total = Social Responsiveness Scale total T-score; SRS-2 SCI T = social communication and interaction T-score; SRS-2 RIRB T= restricted interest and repetitive behavior T-score; PDDBI Soc App T = Pervasive Developmental Disorders Behavior Inventory Social Approach Behaviors T-score; PDDBI REPRIT T = Repetitive, Ritualistic, and Pragmatic Problems Composite T-score; Face Memory SS = NEPSY memory for faces subtask score.

*Inclusion criteria for all participants* included children and their caregivers being English- speaking and children having (if any) stable medication regimens for at least 8 weeks prior to their first visit. *Inclusion criteria for participants with ASD* (n=280) included expert clinical confirmation of DSM-5 criteria diagnosis of ASD (1) as informed by assessments and interviews including the Autism Diagnostic Observation Schedule-2 (ADOS-2; (2)) and the Autism Diagnostic Interview-Revised (ADI-R; (3)); and full scale DAS-II (4) “best IQ” (Full Scale IQ when valid; Full Scale Mental Age/Chronological Age ratio x 100 otherwise) between 60 and 150. *Inclusion criteria for participants with TD* included a Full Scale IQ between 80 and 150; *exclusions* included ADOS score in the ASD range at enrollment, parent report of a biological sibling with ASD, positive screen for emotional or behavioral disorder (CASI-5; (5)), or current treatment for any psychiatric condition. *Exclusions for all participants* included known genetic or neurological conditions; history of non-febrile seizures or current use of anti-epileptic medication; visual, auditory, or sensory-motor impairment that would interfere with eye tracking or standardized assessment; prematurity or pre/perinatal birth injury or brain damage, or severe environmental circumstances impacting brain development.

Additional instruments used in this study also included the Social Responsiveness Scale-2 (SRS-2; (6)); the Pervasive Developmental Disorder Behavioral Inventory (PDDBI; (7)); Vineland-3 Adaptive Scales (Vineland-3; (8)); and A Developmental NEuroPSYchological Assessment, Second Edition (NEPSY-2; (9)): Memory for Faces. A description of instruments and key measures examined are shown in **Table S2**.

#### **Table S2. Summary of Clinical Measures**

| Instrument | Abbr. | Measure | Interpretation |
| --- | --- | --- | --- |
| Autism Diagnostic Obersvation Schedule, Second Edition | ADOS | CSS | Calibrated Severity Scale (Comparison Score) - Higher indicates higher severity of autism symptoms |
|  |  | SA | Social Affect Comparison Score - Higher indicates greater social/affective disability |
|  |  | RRB | Restricted Interests and Repetitive Behaviors Comparison Score - Higher indicates greater RRB |
| Differential Abilities Scales-II | DAS | Full IQ | Full scale IQ from the General Conceptual Ability (GCA) score |
|  |  | Verbal IQ | From the Verbal Cluster - Higher indicates stronger verbal reasoning skills |
|  |  | Nonverbal IQ (NV IQ) | From the Special Non-verbal Composite - Higher indicates greater non-verbal cognitive ability |
| Vineland Adaptive Behavior Scales, Third Edition | VABS3 | ABC | Overall Adaptive Behavior Composite, composed of Communication, Daily Living Skills, and Socialization scales - Higher standard scores indicate presence of greater adaptive behavior skills |
|  |  | Soc SS | Socialization score standard score- Higher indicates more adaptive social ability |
|  |  | Com SS | Communication standard score - Higher indicates greater adaptive communication ability |
| Social Responsiveness Scale, Second Edition | SRS-2, SRS | SCI T | Social Communication and Interaction standardized T-score combines Social Awareness, Cognition, Communication, and Motivation - Higher indicates greater SCI difficulties |
|  |  | RIRB T | Restricted Interests and Repetitive Behaviors T-score - Higher indicates greater presence of RIRB |
| PDD Behavior Inventory | PDDBI | SocApp T | Social Approach T-score - Higher indicates more competent social behaviors |
|  |  | REPRIT T | Repetitive, Ritualistic, and Pragrmatic Problems Composite T-score Higher indicates greater difficulties |
| NEPSY, Second Edition | NEPSY | Face Memory SS | Memory for Faces subtest scaled (M=10, SD=3) score - Higher indicates better encoding of facial features and facial recognition |

Racial profiles of the ASD/TD groups were: 5%/2% Asian, 1%/0% American Indian/Alaska Native, 8%/3% Black/African American, 68%/82% White, 16%/12% Mixed race, and 2%/1% Unreported. Within the ASD/TD group, 9% and 7% of the group, respectively, reported their ethnicities as Hispanic/Latinx.

## Data Acquisition

### Protocol

Room environments for ET sessions were quiet and dimly lit. During set-up, child-friendly animated movies were shown on screen to attract the child’s attention. Positioning was adjusted so children were 65 cm from the screen and centered. No head rest/chin rest was used, with participants’ heads allowed to move freely. Behavioral assistant/s were seated near the child during the session and provided verbal redirections toward the screen when necessary. An experimenter controlled the eye tracking computer and the stimulus presentation computer. Once the eye tracking parameters were set, a series of two calibrations and one validation were performed, followed by the experimental eye tracking paradigms. A calibration was performed approximately every two minutes, involving either five animated targets or a single animation moving in a smooth spiraling trajectory. Before trials a central fixation stimulus (a colorful animation presented concurrently with sound) was displayed (excepting for the Pupillary Light Reflex task).

## Acquisition Metric and Derived Variable Computation

### Data Processing

Core ET data pipelines were written in MATLAB R2017, Perl, and Python. Pipeline components included blink detection, outlier detection, eye-tracking calibration and recalibration, measurements of experimental error, and region-of-interest (ROI) analysis (10–12). ROIs (**Figure S1**) were drawn by hand to be dilated by one visual degree beyond the object edge to mitigate the impact of eye-tracker gaze estimation error.

Static ROI images were used to analyze data from the following tasks: Activity Monitoring (static image trials only), Biological Motion Preference, Static Social Scenes, and Social Interactive (videos divided temporally into three ROI images, each image representing five seconds of the video). Dynamic ROI images were analyzed frame-by-frame for Activity Monitoring (video trials only). ROI derived variables (e.g. %Face, %Activity) were calculated as the amount of time spent looking at the ROI divided by the total amount of validly acquired ET data on a per trial basis. Data from all valid trials were averaged to obtain primary and secondary derived variables for each timepoint.

Pupillary Light Reflex data was downsampled to 50hz and analyzed via semi-manual coding through additional programs based on (13,14). The onset and maximal constriction of each PLR trial was automatically detected and each trial was manually checked, and corrected if needed, to ensure accurate detection of the relevant waveform features.

#### **Figure S1. Region-of-interest (ROI) examples for ActivityMonitoring (AM), Biomotion (BM), SocialInteractive (SI), and StaticScenes (SS) tasks.** Black bars over eyes did not appear in stimuli shown to participants.


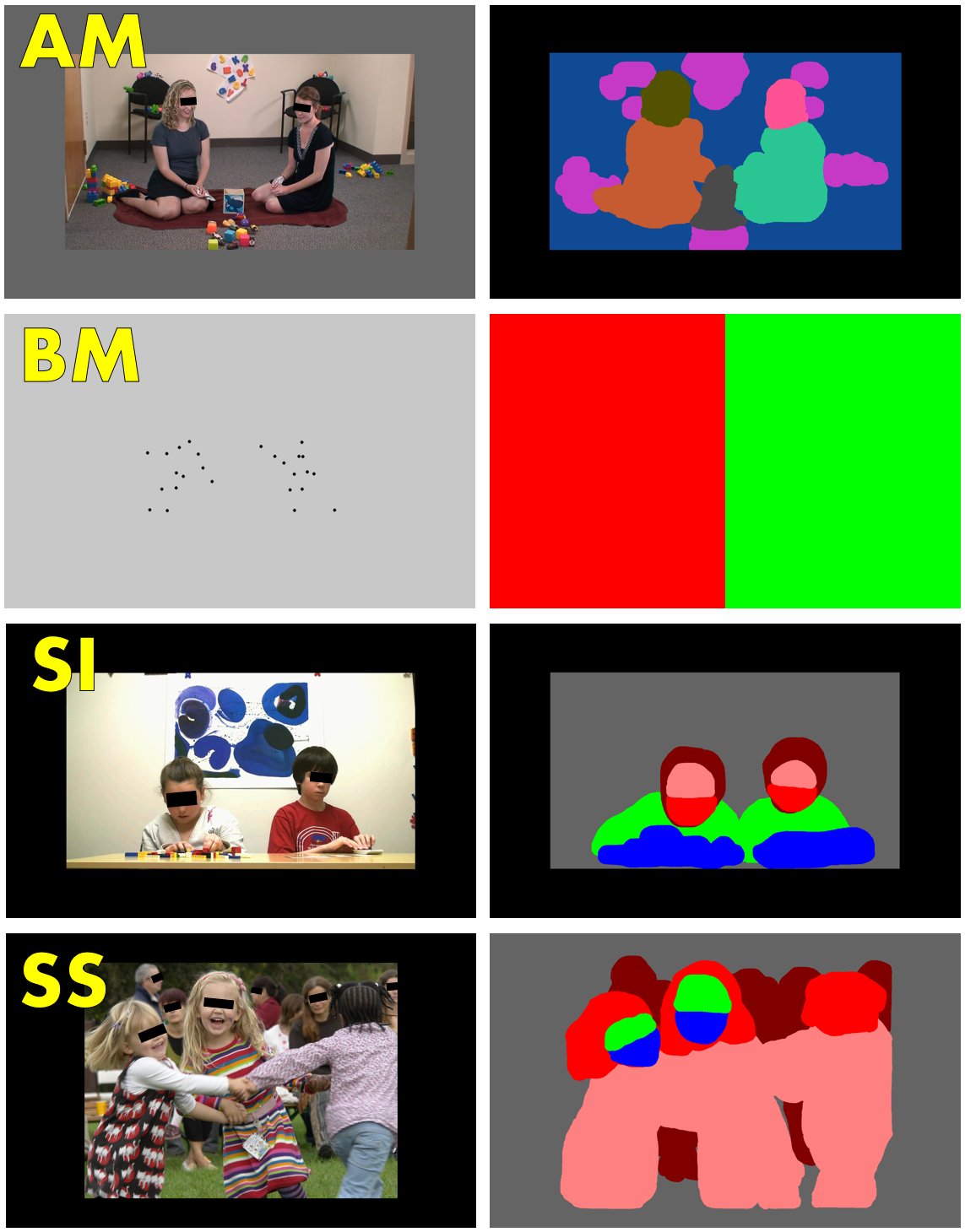


### Data Validity Criteria

For ActivityMonitoring, StaticScenes, SocialInteractive,and Biomotion, trials were considered valid if (1) non-artifactual eye tracking data were collected for at least 50% of the trial presentation time from at least one eye, and (2) calibration error (more specifically, “calibration uncertainty”, see Calibration Quality notes, below) was less than 2.5 visual degrees. For PLR, trials were considered valid if (1) at least one eye provided usable pupil size estimates; (2) baseline pupil amplitude was temporally unbroken and stable; (3) latency and minimum pupil amplitude were reliably identified; and (4) average gaze position was within 5 degrees of the center of the screen.

Primary acquisition metrics included %Valid Trials, the number of valid trials obtained in a task relative to the maximum number of delivered trials; %Valid Data, the amount of eye-tracking data obtained in a trial divided by the total stimulus presentation time of those trials, averaged across both valid and invalid trials; and Cal Error, the amount of calibration uncertainty/error in trials, averaged across all valid trials. %Valid Data was bounded between 0 and 100%, whereas Cal Error was effectively unbounded above 0 (in cases of calibration degeneracy), and so while %Valid Data was reported for both valid and invalid trials (thus providing a measure of overall attention more independent of the subset of trials that were valid), Cal Error was only reported for valid trials (thus providing a measure of the calibration error/uncertainty of retained trials).

### Calibration Quality

Calibration targets interleaved periodically throughout ET sessions (“internal calibrations”, as compared to “hardware calibrations” applied directly to incoming gaze location estimates by the eye tracker hardware) were used for computation of calibration uncertainty and posthoc analytical adjustment of scanpaths to compensate for drift in calibration quality over time. Calibration uncertainty was calculated as the mean absolute distance between scanpath trajectories computed using all internal calibration points over the entire ET session (the “static” calibrated trajectory) and the scanpath trajectories computed by either the temporally nearest set of calibration points (the “nearest neighbor” calibrated trajectory) or the raw gaze locations reported by ET hardware (the “raw” trajectory). The minimum distance of either static-nearest neighbor or static-raw calibration determined whether nearest-neighbor gaze coordinates or raw gaze coordinates were used in subsequent computation for that trial. All procedures were automated, with no subjective human involvement. Comparisons between this error and errors computed via adjacent but non-overlapping internal calibrations suggested that effective calibration error, defined in the traditional sense of distance between gaze estimates and actual point of regard, was less than 70% of gaze uncertainty. Details and rationale regarding these methods can be found in (10). For ease of terminology, we refer to calibration uncertainty as “calibration error” throughout this document and in the main text (without the 70% error adjustment).

## Experimental Tasks

Overall task structure is summarized in **Table S3**. Experiments were developed in order to address the specific needs of biomarker development as compared to experimentation designed to examine questions regarding mechanism. In order to optimize evaluation of stability/test-retest properties, once an ordering of experimentation was selected for a participant at baseline, the participant was shown stimuli in the same order at each subsequent timepoint. In addition, while the actual experimental task was counterbalanced across participants, many experiments were delivered in a fixed stimuli order so as to maintain comparability with prior studies and to reduce intersubject variability. Unique properties related to counterbalancing for each experiment are described in the corresponding sections below.

Each experiment was delivered in blocks (a contiguous run of stimuli from one particular experiment). These blocks were interwoven with one another so as to reduce participant fatigue and increase participant engagement in the ET session as a whole, while still enabling examination of biomarker stabilization effects across the repeated intrablock structure. The Pupillary Light Reflex task was an exception to this structure, with each “block” consisting of a single trial, and PLR trials dispersed across the entire ET session (rather than in block form).

Experimental stimuli were calibrated to maintain comparability to source experimentation in terms of the size of presentation in visual angles.

#### **Table S3. ET Task protocol parameters and and dependent variables (DVs)**


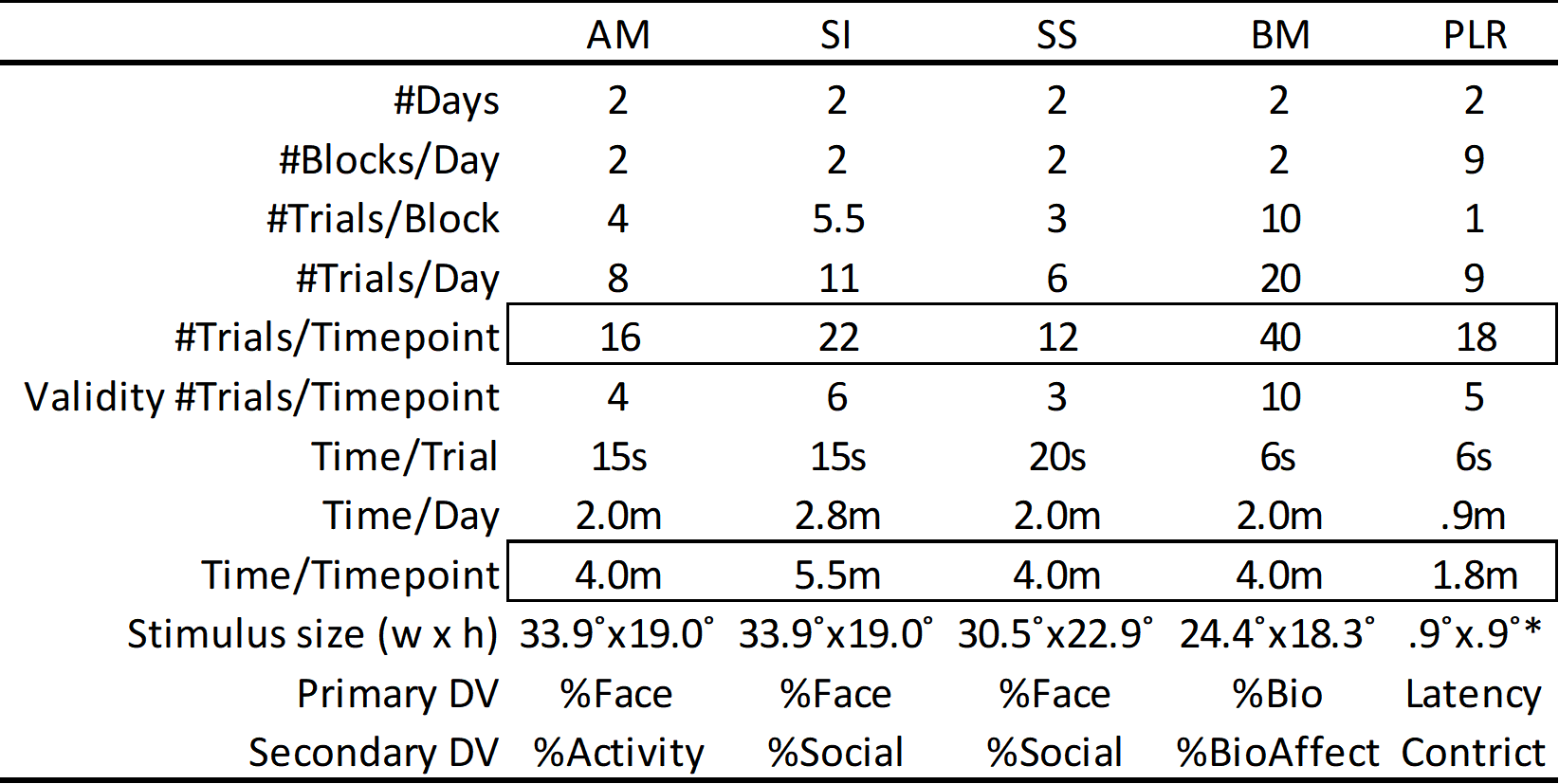


Table Key: AM=Activity Monitoring Task; SI=Social Interactive Task; SS=Static Social Scenes Task; BM=Biological Motion Preference Task; PLR=Pupillary Light Reflex Task; DV=Dependent Variable; *For PLR this is the size of the central stimulus, however the flash encompasses the entire screen i.e. 45.7°x28.6°

### Activity Monitoring Task (ActivityMonitoring)

*A) Background:* Toddlers with ASD attend less to activities and faces compared to controls, with atypical gaze patterns associated with IQ and autism severity (15,16). Recent studies show that decreased looking at faces, but not activities, persists even in later childhood and in adults with ASD (17,18). *B) Procedure*: Following a central fixation, children were presented with multiple activity monitoring trials as developed in (16). Stimuli were images or videos of two actresses engaged in simple child-appropriate games with colorful toys in the background. Videos included audio of task-relevant conversation between actresses; images included a classical music soundtrack. Embedded conditions included the *gaze* of actresses (directed at each other or towards the activity) and *movement* (whether stimuli were static images or dynamic videos). Stimuli were shown in a fixed order but presented horizontally flipped for half of participants. There were 8 sets of actresses, 8 sets of backgrounds, and 8 sets of scripts, ordered to minimize sequential repeats. Conditions of *gaze* and *movement* were ordered for within-subject balance and a maximum of two sequential repeats of any level. *C) Primary Dependent Variable:* % of time spent looking at heads and faces of actresses (%Face). *Secondary Dependent Variable: %* of time looking at activities and the activity area (%Activity). *D) Hypotheses: %*Face (Primary): ASD < TD; %Activity (Secondary): ASD <TD. We hypothesized DVs would be positively associated with language ability and social abilities. These hypotheses were driven by information available at the inception of the study based on toddlers with and without ASD.

### Social Interactive Task (SocialInteractive)

*A) Background:* Chevallier and colleagues in (19) used an “interactive social VE (Visual Exploration) task” to examine looking patterns in 12-year-old children with ASD (*n* = 59) and TD children (*n* = 22). This task depicted children playing silently with objects and each other. The authors found that children with ASD looked less at faces than TD children. *B) Procedure*: Following a central fixation, children were presented with silent videos of interactive social trials as described in (19). Embedded conditions included the nature of play: parallel (children play on their own with toys) or cooperative (children play with each other with toys). As per (19)*,* stimuli were presented in the same, prefixed order for all participants. Conditions of parallel and cooperative play alternated with one another in the presentation sequence. Unique child play pairs (11 possible) were each shown twice (once in each play condition), with no child-pair sequential repeats over trials, and each unique play pair shown before any play pair was shown again. *C) Primary Dependent Variable:* % time spent looking at heads and faces of child actors (%Face); *Secondary Dependent Variable:* % time spent looking at social aspects of the scene, including head, body, and activity (%Social). *D) Hypotheses:* %Face: ASD < TD; %Social: ASD < TD. %Social was the original primary variable and %Face a secondary variable at the onset of the Feasibility study, but the two were swapped in their designation post-Feasibility in the Main Study.

### Static Social Scenes Task (StaticScenes)

*A) Background:* Prior studies have found that individuals with ASD show atypical gaze patterns when presented with static images of faces and scenes involving people (20,21). *B) Procedure*: Following a central fixation, children were presented with static images depicting children engaged in natural, everyday, solitary or socially-interactive activities (with other children or adults). These scenes were presented simultaneously with a range of musical soundtracks and were developed as part of the EU-AIMS consortium project (<https://www.eu-aims.eu/>). This task was interleaved (alternating trials) with another task, the “Visual Search task” (22,23), which was deprioritized in the Main Study but retained to keep the Static Social Scene trial structure intact (see (24)). Six unique images were shown to participants in a fixed order on the first day of each timepoint, and, on the second day, the images shown again except horizontally flipped. The order of horizontal orientation (flipped or not flipped) was counterbalanced across participants. *C) Primary Dependent Variable:* % time spent looking at faces of any person in the image (%Face); *Secondary Dependent Variable:* % time spent looking at social aspects of the scene, including heads, bodies, and activities (%Social). *D) Hypotheses:* %Face: ASD < TD; %Social: ASD < TD. These hypotheses were based on reports of diminished focus on faces in complex naturalistic images in ASD (25).

### Oculomotor Index of Gaze to Human Faces (OMI)

After the conclusion of the ABC-CT Feasibility Study (which involved children with ASD (N=25) and TD (N=26), see **Table S4**), ET primary and secondary variables at the aggregate (participant) level were analyzed using principal component analyses. These analyses revealed a primary (first) principal component (**Table S5**) explaining 31.3% of variance, dominated by positive contributions of %Face in ActivityMonitoring, SocialInteractive, and StaticScenes tasks. Note these analyses also included overall data rate acquisition, %Valid Data (averaged across all tasks), and percentage of time looking at faces in an implicit Visual Search (VS) task (removed from analytic consideration subsequent to the Feasibility Study). See (24) for additional details regarding the transition from the ABC-CT Feasibility Study to the ABC-CT Main Study.

#### **Table S4. Feasibility ET Participant Characteristics**

| **Characteristic** | **TD** | **ASD** |
| --- | --- | --- |
| N (Males:Females) | 26 (17:9) | 25 (20:1) |
| Age in Years [M (SD)] | 6.6 (2.0) | 7.8 (2.3) |
| VIQ [M (SD)] | 125.0 (18.1) | 89.6 (26.6) |
| NVIQ [M (SD)] | 117.4 (11.7) | 95.8 (23.1) |
| ADOS CSS [M (SD)] | 1.0 (.4) | 7.8 (1.6) |

#### **Table S5. Feasibility ET 1^st^ Principal Component Weights**

| **Task** | **Variable** | **Weight** |
| --- | --- | --- |
| **AM** | **%Face** | **.759** |
| **SS** | **%Face** | **.748** |
| **SI** | **%Face** | **.732** |
| Overall | %Valid Data | .541 |
| PLR | Latency | -.124 |
| PLR | Constrict | .158 |
| BM | %Bio | .451 |
| VS | %Face | .547 |

Because the weights of these components were largely comparable, they were aggregated into a single composite biomarker, the Oculomotor Index of Gaze to Human Faces (OMI), as the simple average of %Face across the three tasks of ActivityMonitoring, SocialInteractive, and StaticScenes.

### Biological Motion Preference Task (Biomotion)

*A) Background:* Adults quickly and spontaneously recognize human figures in point-light displays (PLDs) consisting of a small number of illuminated dots moving like the joints of a person walking or engaging in other activities (26). Preferences for biological motion (biomotion) have been found not only in human newborns (27) but in other species as well (28). Yet, individuals with ASD have shown difficulties recognizing biological motion (29–32) and toddlers, children, and adults with ASD also show atypical preferences for biological motion versus control motions (33–35). *B) Procedure*: Following a central fixation, children were presented with two animated PLDs side-by-side, one of which was a biomotion target and the other being a scrambled or rotating perceptual control (3-5 s). Human biological motion included primitive motor, affective, communicative, tool-oriented, or goal-oriented movements from the CMU motion capture database (36). Rotating controls used the dominant frequency of hip motion from the human biological motion, as determined by autocorrelation, to define the rotational speed. Scrambled controls were phase-scrambled at each point light by a random assignment of phase offsets evenly sampled across the dominant hip movement frequency and random shuffling of point z-coordinates (head-to-heel axis). Embedded conditions included the control type (scrambled or rotational) and the orientation (horizontally flipped or not flipped), as well as the specific content class of the human biological motion (e.g. affective). Stimuli content (i.e. the base human biological motion video) were shown in a fixed order, with control type and orientation counterbalanced across participants. *C) Primary Dependent Variable (DV):* % of time spent looking at the biological motion relative to time spent looking at either the biological motion or the control stimulus (%Bio). *Secondary Dependent Variable (DV):* % of time spent looking at biological motion relative to biological motion or control stimulus looking for affective stimuli only (%BioAffect). *D) Hypotheses:* %Bio, %BioAffect: ASD < TD.

### Pupillary Light Reflex Task (PLR)

*A) Background:* The Pupillary light response (PLR) is a measure of autonomic nervous system function that is not under volitional control. A bright flash of light causes the pupil to rapidly constrict and then slowly re-dilate. The dynamics of this constriction and dilation can be used to index action of the central nervous system in a non-invasive fashion with good signal to noise characteristics. Subjects’ pupillary responses to white flashes of light can be measured using non-invasive eye-tracking technology. School-age children and adults with ASD exhibit longer PLR latency, reduced constriction amplitude, and shorter redilation time than TD children in response to flashes of light, suggesting reduced parasympathetic modulation (13,37). By contrast, in high-risk infant siblings, increased relative pupil constriction in infancy is associated with higher ASD symptom severity at 3 years of age (38). *B) Procedure*: The video stimulus began with a small, central, light blue object which continuously rotated while expanding and contracting, set against a black background (14). At a specified time in presentation (onset time = 65, 71, or 78 frames at 30 fps), the background would flash to white for 4 frames ~ 132ms. The video with the black background would then continue until completion. Audio involved a synthetic, repeating, pulse sound. The onset time of the flash was counterbalanced across participants, with an equal number of onset time presentations maintained throughout the experiment, and no two consecutive trials using the same onset time. *C) Primary Dependent Variable (DV):* Latency to minimum pupil size acceleration (Latency). *Secondary Dependent Variable (DV):* relative pupil constriction (Constrict). Hypotheses were based on results by (13,14).

## Analytic Plan

### Correlation Method Rationale

Spearman’s was selected over Pearson’s correlation to accommodate potential deviations from normality (especially outliers), and over Kendall’s Tau for more intuitive comparability with extant literature (which traditionally uses either Pearson’s or Spearman’s correlations). In practice, significance patterns were largely unaffected by this choice, e.g. see Clinical Correlations in **Supplemental Tables S13a** vs.**S13a1** and **S13a2**.

### Acquisition, Group Discrimination, and Clinical Correlation Control Variables

Age was controlled for in analyses due to relationships between age and ET biomarkers identified in the TD sample (**Table S13b**). IQ was controlled for due to the presence of significant IQ differences between ASD and TD groups (**Table 1**). %Valid Data was selected as a general measure of data quality, correlated with other acquisition metrics (**Table S10**), but demonstrated some of the largest relationships with child characteristics (**Table S8**) as well as ET biomarkers (**Table S9**). %Valid Data was used as a control in group discrimination (**Tables 2, S12ab**) and ET-clinical correlation (**Tables 2, S13ab**) analyses to provide greater information regarding the unique properties of ET biomarkers above and beyond those associated with acquisition metrics.

# Results

## Acquisition

#### **Table S6a. Signal Acquisition and Validity at T1.** Number of participants (%) with acquired and valid signal by group and ET task at Baseline (T1). Chi-square test on valid signal differences between sites.
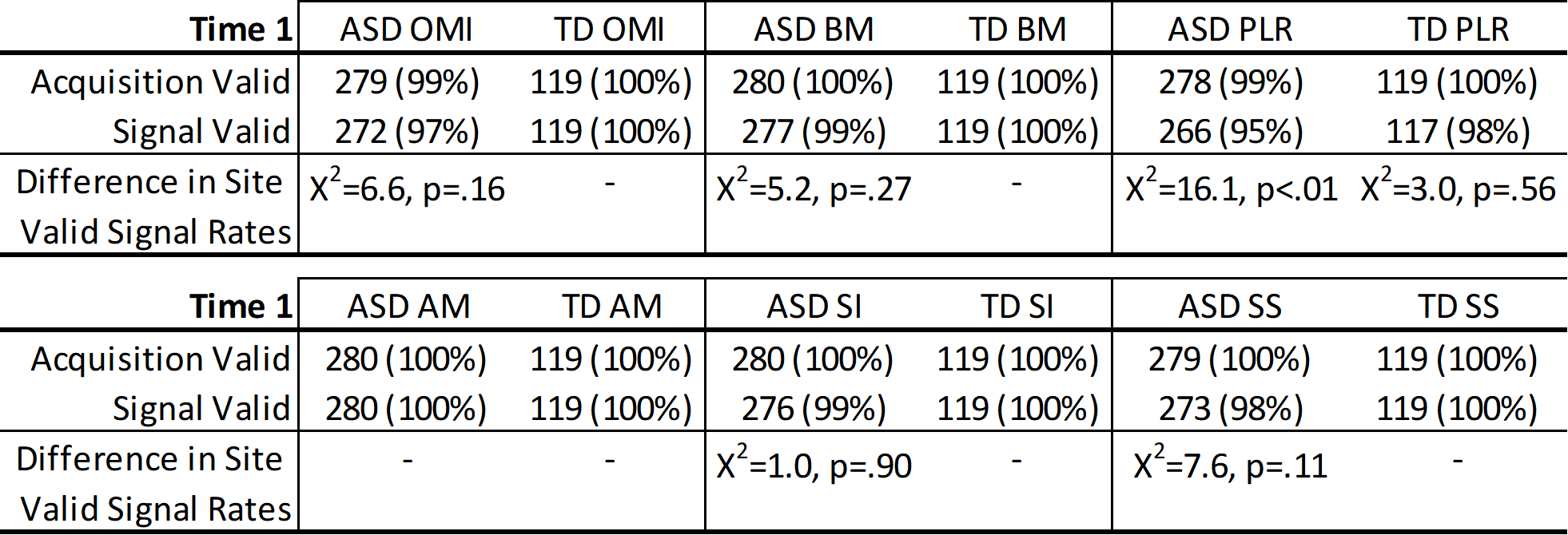


Table Key: OMI=Oculomotor Index of Gaze to Human Faces; BM=Biological Motion Preference; PLR=Pupilary Light Reflex; AM=Activity Monitoring; SI=Social Interactive; SS=Static Scenes; Acquisition Valid = participant generated ET data for some portion of the assay; Signal Valid = valid signal for primary DV (meeting all quality control criteria for admission of data); Difference in Site Valid Signal Rates = Pearson’s Chi-Squared test for site differences in valid signal (consistent with Monte Carlo simulation and unable to be computed for 100% data validity)

#### **Table S6b. Signal Acquisition and Validity at Time 2.** Number of participants (%) with acquired and valid signal by group and ET task at T2. Chi-square test on valid signal differences between sites.

**
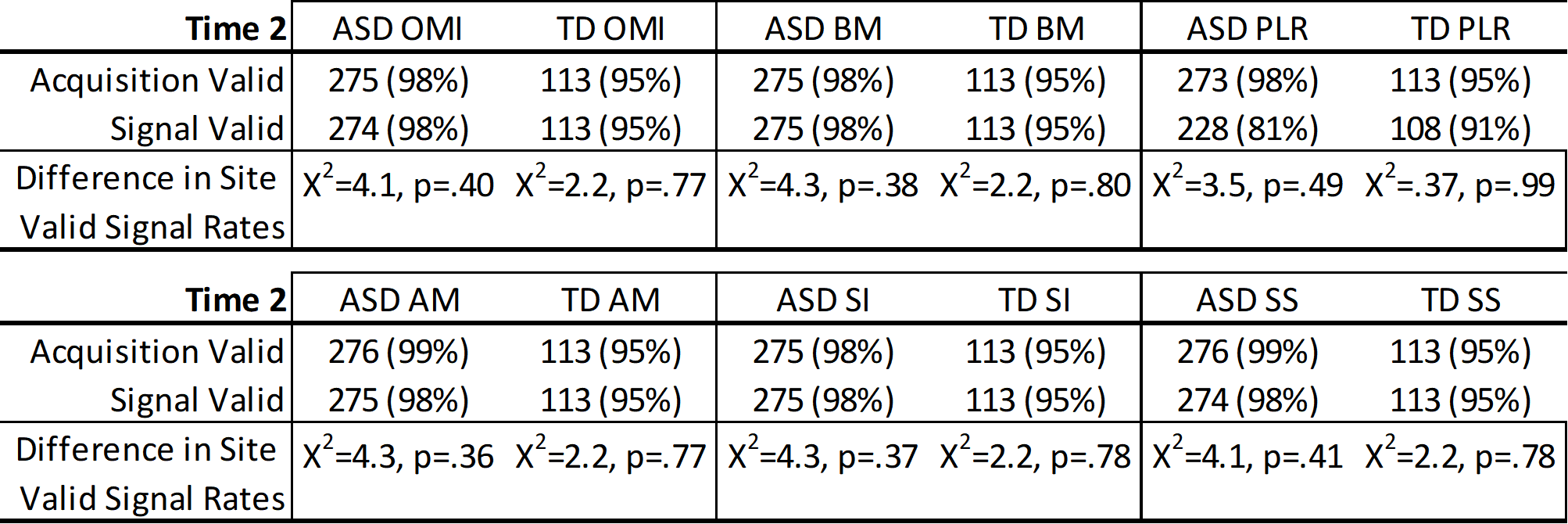
**

Table Key: OMI=Oculomotor Index of Gaze to Human Faces; BM=Biological Motion Preference; PLR=Pupilary Light Reflex; AM=Activity Monitoring; SI=Social Interactive; SS=Static Scenes; Acquisition Valid = participant generated ET data for some portion of the assay; Signal Valid = valid signal for primary DV (meeting all quality control criteria for admission of data); Difference in Site Valid Signal Rates = Pearson’s Chi-Squared test for site differences in valid signal.

#### **Table S7. Time 1 Group Discrimination for ET task acquisition metrics.** Group means (standard deviations), effect sizes, and ANOVA/ANCOVA tests are presented unadjusted and controlling for age, IQ, and site.


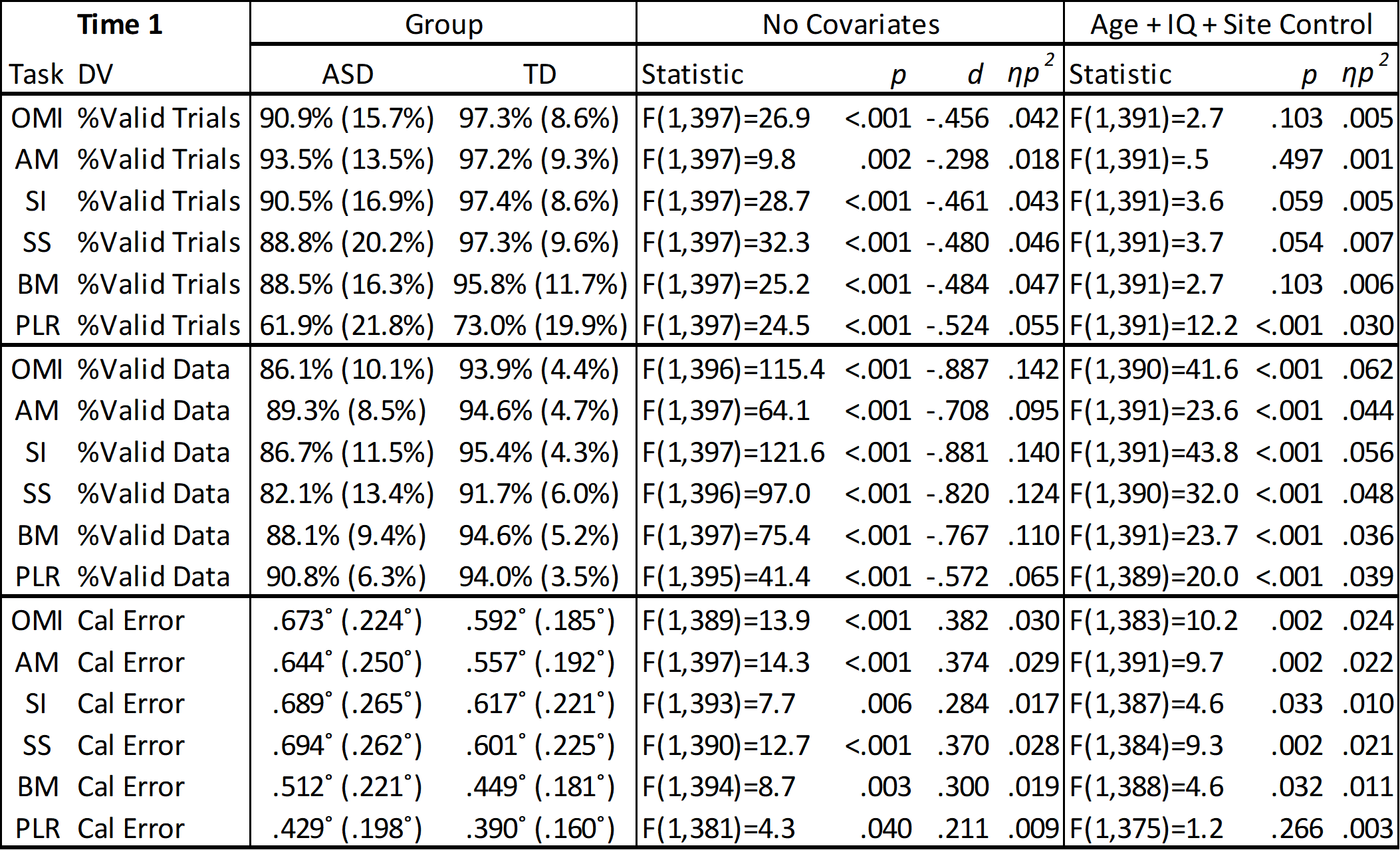


Table Key: DV = dependent variable; Age = participant age; IQ = full scale IQ; Site = data collection site.

#### **Table S8. Spearman Correlations between ET acquisition metrics and child characteristics at Time 1.** Underlined correlations are significant at *p*<.05 even after controlling for Age and Full Scale IQ.
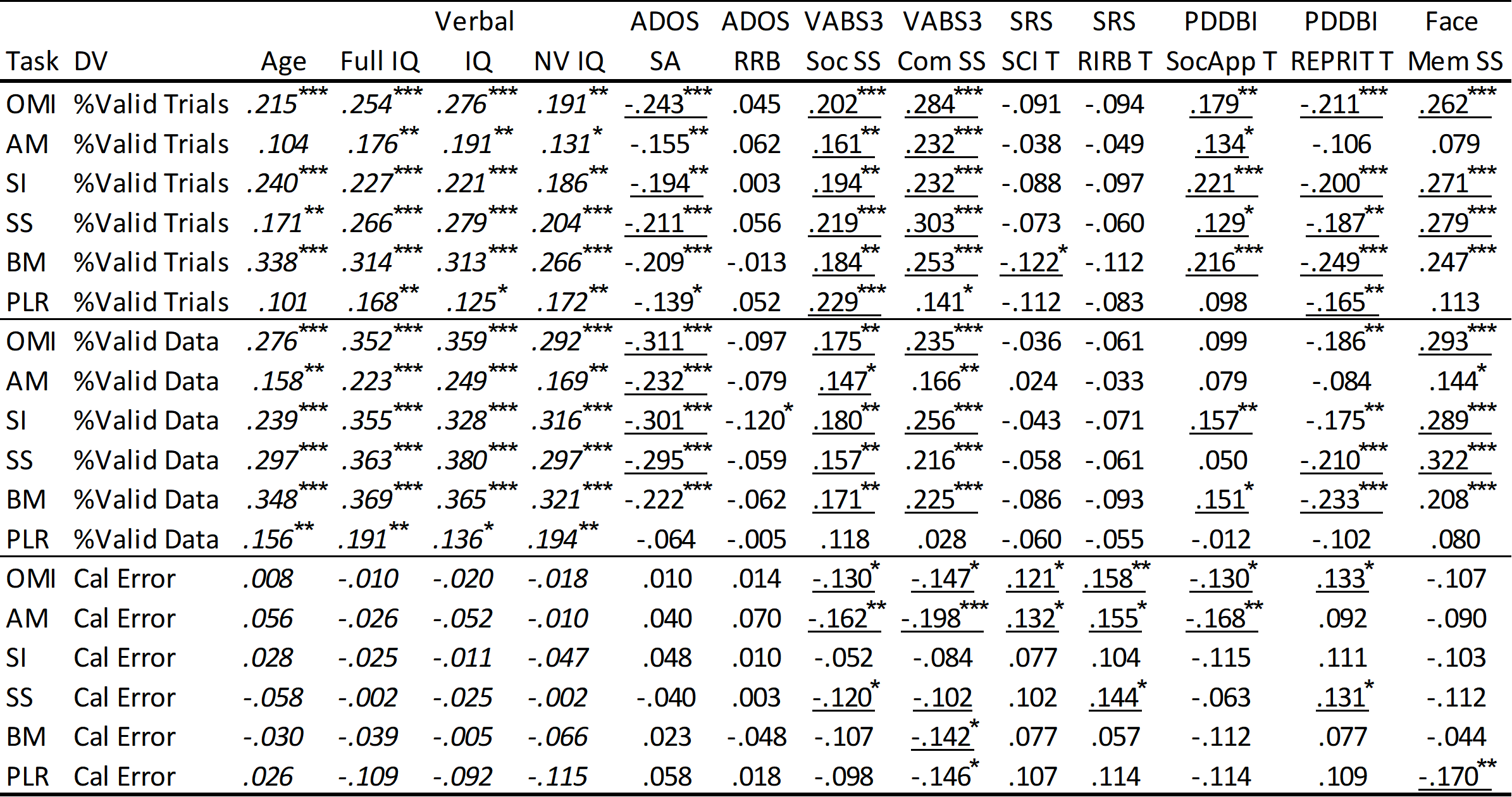


Table Key: OMI=Oculomotor Index of Gaze to Human Faces; AM=Activity Monitoring; SI=Social Interactive; SS=Static Scenes; BM=Biological Motion Preference; PLR=Pupilary Light Reflex; Full IQ = DAS Full Scale IQ; NV IQ = DAS Nonverbal IQ; ADOS SA = Autism Diagnostic Observation Schedule social affect comparison score; ADOS RRB = restricted interests and repetitive behavior comparison score; VABS3 Soc = Vineland Adaptive Behavior Scales adaptive behavior socialization standard score; VABS3 Com = communication standard score; SRS-2 SCI = Social Responsiveness Scale social communication and interaction T-score; SRS-2 RRB = restricted interest and repetitive behavior T-score; PDDBI SoccApp T = Pervasive Developmental Disorders Behavior Inventory Social Approach Behaviors T-score; PDDBI REPRIT = Repetitive, Ritualistic, and Pragmatic Problems Composite T-score; Face Mem SS = NEPSY memory for faces subtask score. ^*^*p*<.05; ^**^*p*<.01; ^***^*p*<.001. Underlined cells are significant even after controlling for Age and Full Scale IQ. *Italicized* cells cannot be controlled for these variables due to collinearity.

#### **Table S9. Spearman Correlations between ET biomarkers and ET acquisition metrics in the ASD group at Time 1.** Underlined correlations are significant at *p*<.05 even after controlling for Age and Full Scale IQ.


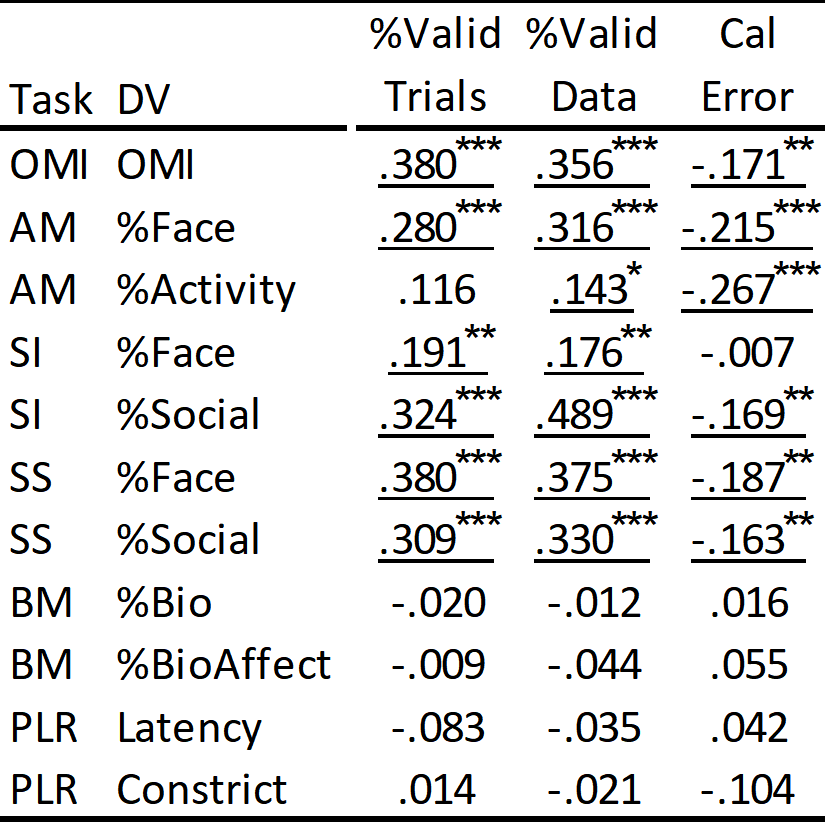


Table Key: OMI=Oculomotor Index of Gaze to Human Faces; AM=Activity Monitoring; SI=Social Interactive; SS=Static Scenes; BM=Biological Motion Preference; PLR=Pupilary Light Reflex; ^*^*p*<.05; ^**^*p*<.01; ^***^*p*<.001. All significant findings remained so even after controlling for Age and Full Scale IQ (underlined).

#### **Table S10. Spearman Correlations among ET acquisition metrics in the ASD group at Time 1.** Underlined correlations are significant at *p*<.05 even after controlling for Age and Full Scale IQ.


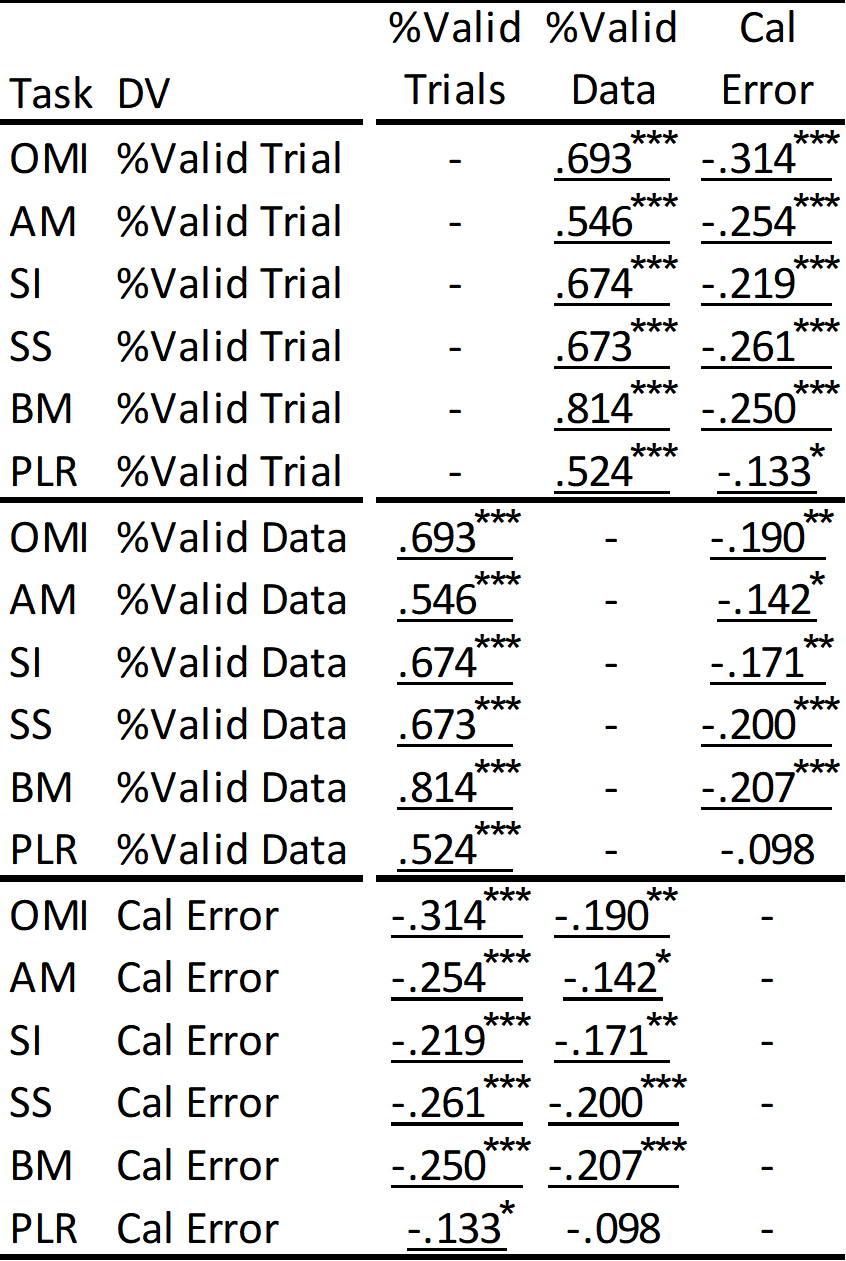


Table Key: OMI=Oculomotor Index of Gaze to Human Faces; AM=Activity Monitoring; SI=Social Interactive; SS=Static Scenes; BM=Biological Motion Preference; PLR=Pupilary Light Reflex; ^*^*p*<.05; ^**^*p*<.01; ^***^*p*<.001. All significant findings remained so even after controlling for Age and Full Scale IQ (underlined).

##

## Construct Validity

As a stronger benchmark for “chance-level looking at faces” (for ActivityMonitoring, SocialInteractive, and StaticScenes tasks), we used a computational model of visual attention designed to represent low-level visual saliency. The computational model used was based on one of the most well-studied models used for saliency computation (39), but was extended for motion saliency calculation in (40,41) so as to be more applicable to salient visual features present in videos. Saliency calculations computed from aggregated conspicuity maps calculated across feature modalities were scaled to the size of visual content area of stimuli presentation (occupying a smaller subset of the entire display) and corresponding energies (i.e. total saliency signal) within face ROIs extracted as a ratio against the total scene energy (here we refer to this as %Face_saliency_). For ActivityMonitoring, %Face_saliency_ was 8.5%, with one sample *t*(118)=24.6, *p*<.001, *d*=2.25; for SocialInteractive %Face_saliency_=18.9%, *t*(118)=13.0, *p*<.001, *d*=1.19; for StaticScenes %Face_saliency_=9.4%, *t*(118)=34.8, *p*<.001, *d*=3.19. These results, while notably weaker than those computed based on simple completely random gaze patterns limited to the screen display (as shown in **Table 2,** **Table S11a**), were still highly significant, meeting expected criteria for construct validity based on TD children. Because there is no agreed-upon method for saliency computation, we have retained the weaker, more basic, but more easily replicated chance-based-on-area construct tests in primary tables in this work, but acknowledge the need for ongoing research into appropriate benchmark methods.

Similar to construct validity in the TD group (**Table 2,** **Table S11a**), all tasks induced above-random chance performance in the ASD group (**Table S11b**). Using the selected computational model baseline of %Face_saliency_, in ASD we found, for ActivityMonitoring, *t*(279)=19.7, *p*<.001, *d*=1.18; for SocialInteractive, *t*(279)=8.2, p<.001, *d*=.49; for StaticScenes, *t*(279)=35.0, *p*<.001, *d*=2.09. Notably, while both Social Interactive constructs and Biological Motion preference constructs, measured with a saliency model baseline, were statistically significant in ASD, their effect sizes were modest compared to other tasks.

#### **Table S11a. Construct Validity for each ET task based on TD participants at T1.**


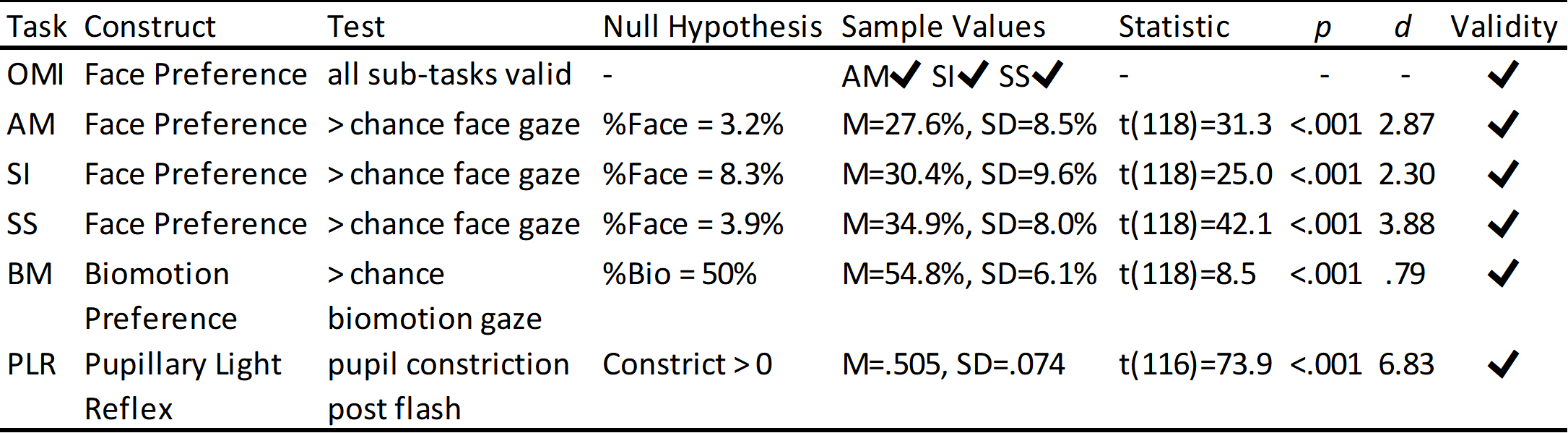
Table Key: OMI=Oculomotor Index of Gaze to Human Faces; AM=Activity Monitoring; SI=Social Interactive; SS=Static Scenes; BM=Biological Motion Preference; PLR=Pupilary Light Reflex; Task = ET task; Construct = hypothesized construct under investigation; Test = how the construct is tested; Null Hypothesis = formal definition of the construct validity test; Sample Values = TD performance on null hypothesis variable at T1.

#### **Table S11b. Construct Validity for each ET task based on ASD participants at T1**.


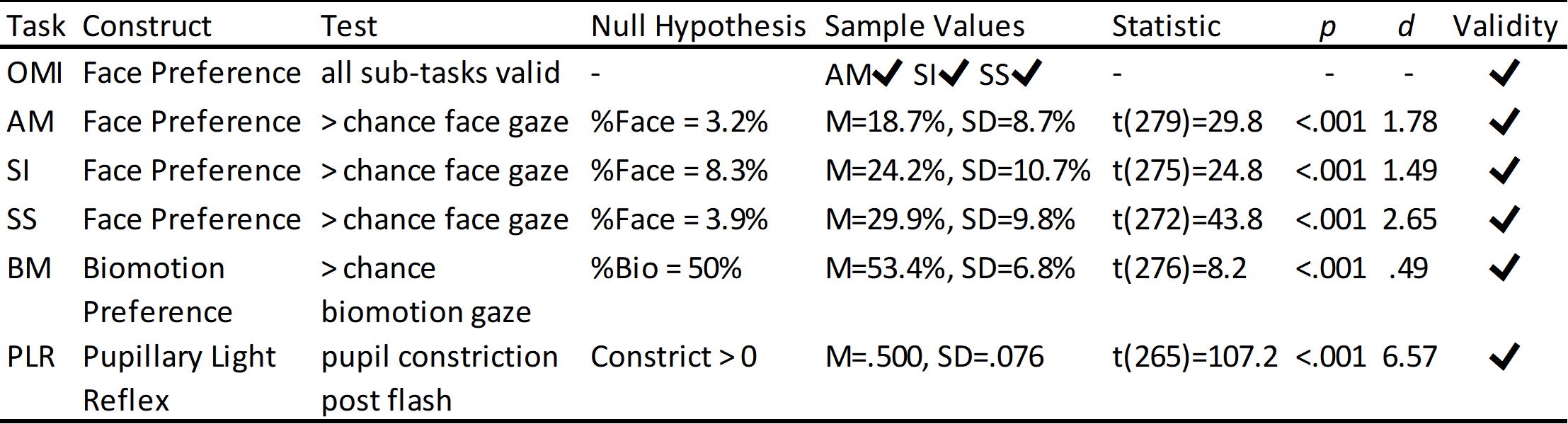
Table Key: OMI=Oculomotor Index of Gaze to Human Faces; AM=Activity Monitoring; SI=Social Interactive; SS=Static Scenes; BM=Biological Motion Preference; PLR=Pupilary Light Reflex; Task = ET task; Construct = hypothesized construct under investigation; Test = how the construct is tested; Null Hypothesis = formal definition of the construct validity test; Sample Values = TD performance on null hypothesis variable at T1.

## Six-week Stability

#### **Figure S2. Six-week stability (T1 to T2) of the ASD group for the primary dependent variables of ActivityMonitoring (AM), SocialInteractive (SI), and StaticScenes (SS) %Face.** Line is identity (slope=1).
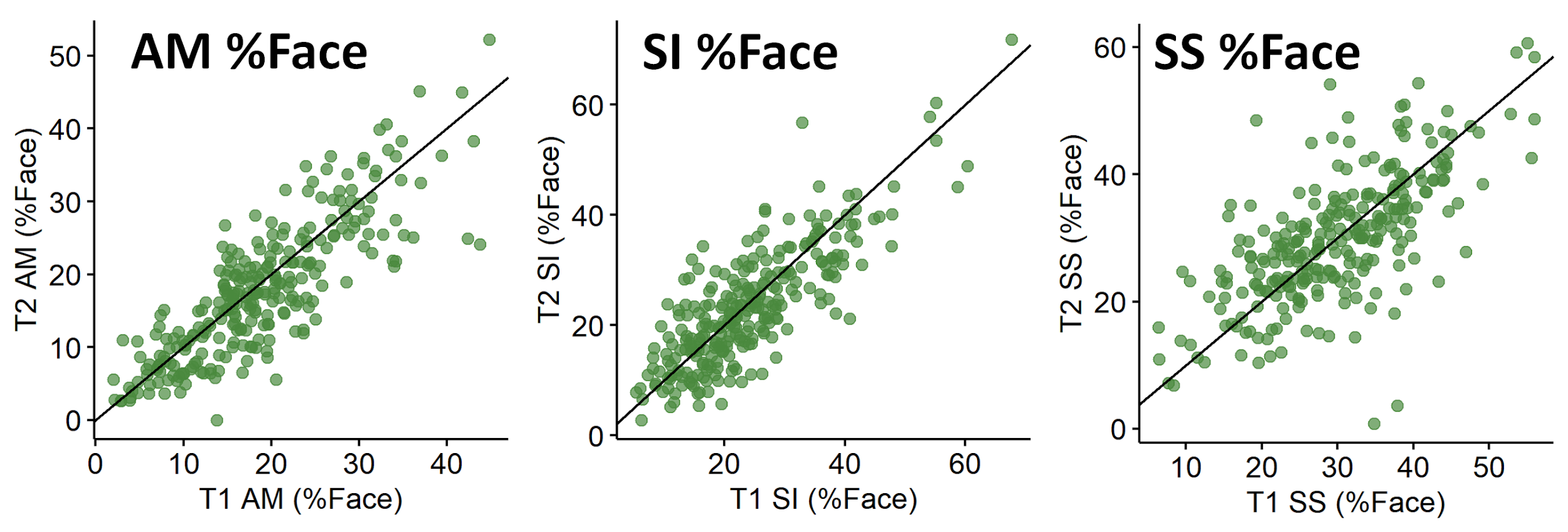


## Group Discrimination

#### **Figure S3. Boxplots (top) and Histograms (bottom) of ASD and TD (left to right) ActivityMonitoring (AM), SocialInteractive (SI), and StaticScenes (SS) %Face Biomarkers.****
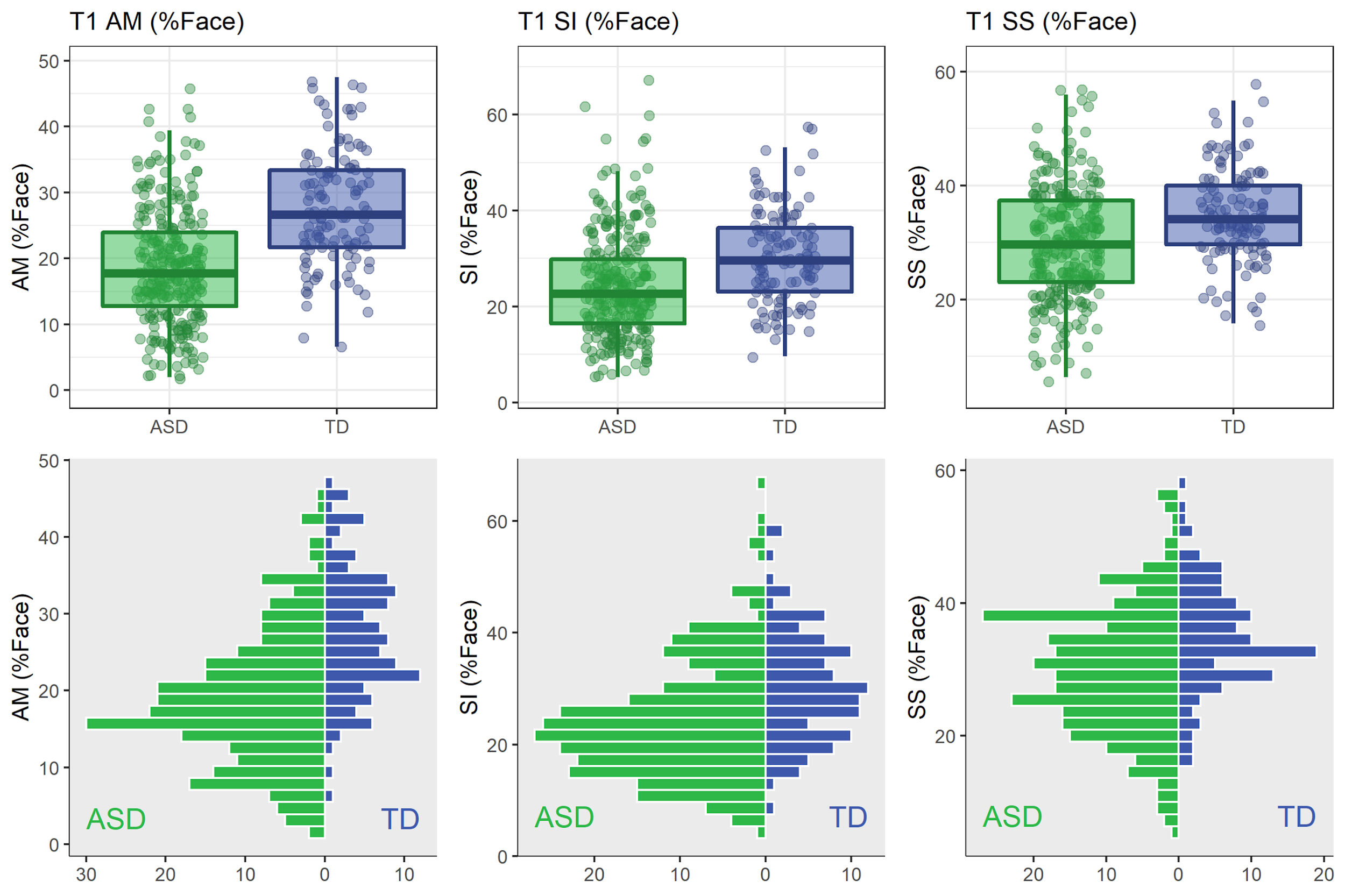
**

#### **Table S12a. T1 Group Discrimination for primary and secondary variables**. Group means (standard deviations), effect sizes, and ANOVA/ANCOVA tests are presented unadjusted and controlling for age, IQ, site, and %Valid Data.


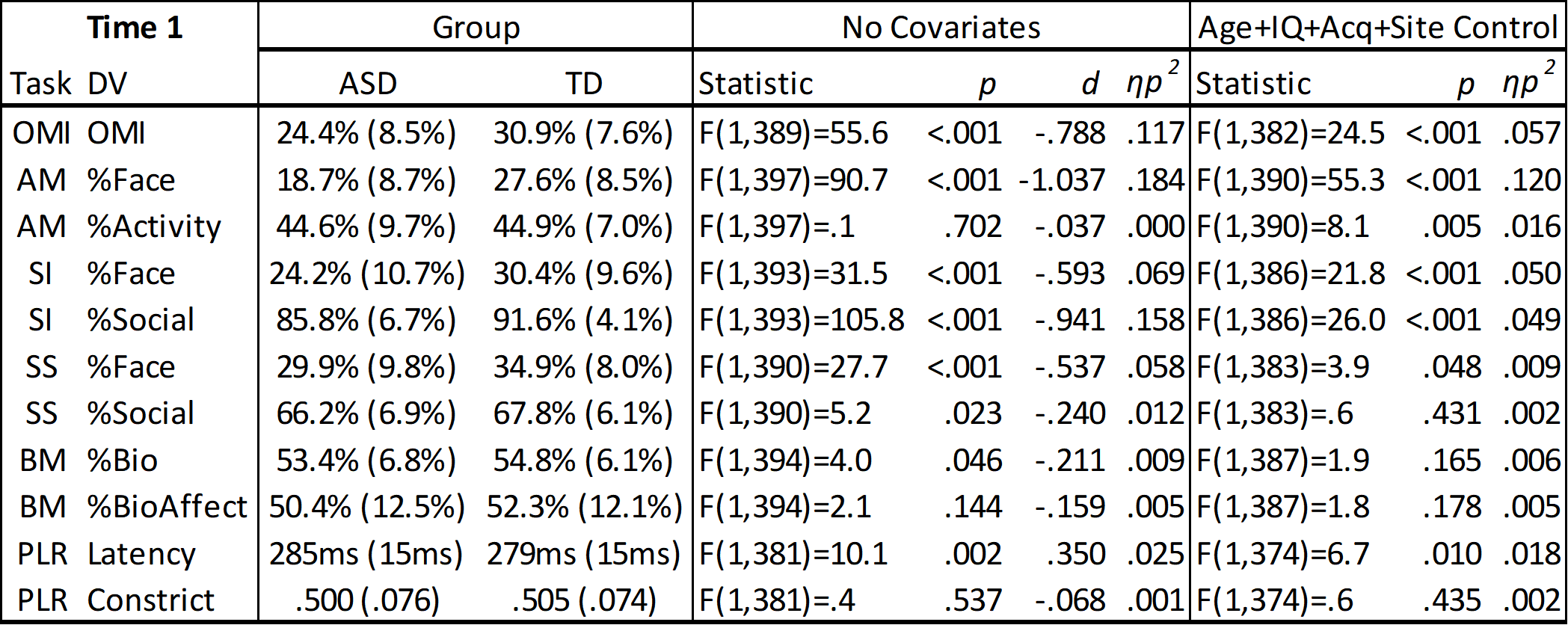


Table Key: OMI=Oculomotor Index of Gaze to Human Faces; AM=Activity Monitoring; SI=Social Interactive; SS=Static Scenes; BM=Biological Motion Preference; PLR=Pupilary Light Reflex; DV = dependent variable; Age = participant age; IQ = Full Scale IQ; Acq = %Valid data collection rate; Site = data collection site.

#### **Table S12b. Time 2 Group Discrimination for primary and secondary variables**. Group means (standard deviations), effect sizes, and ANOVA/ANCOVA tests are presented unadjusted and controlling for age, IQ, site, and %Valid Data. Gaze pattern variables are shown on the top, data validity variables on bottom. **
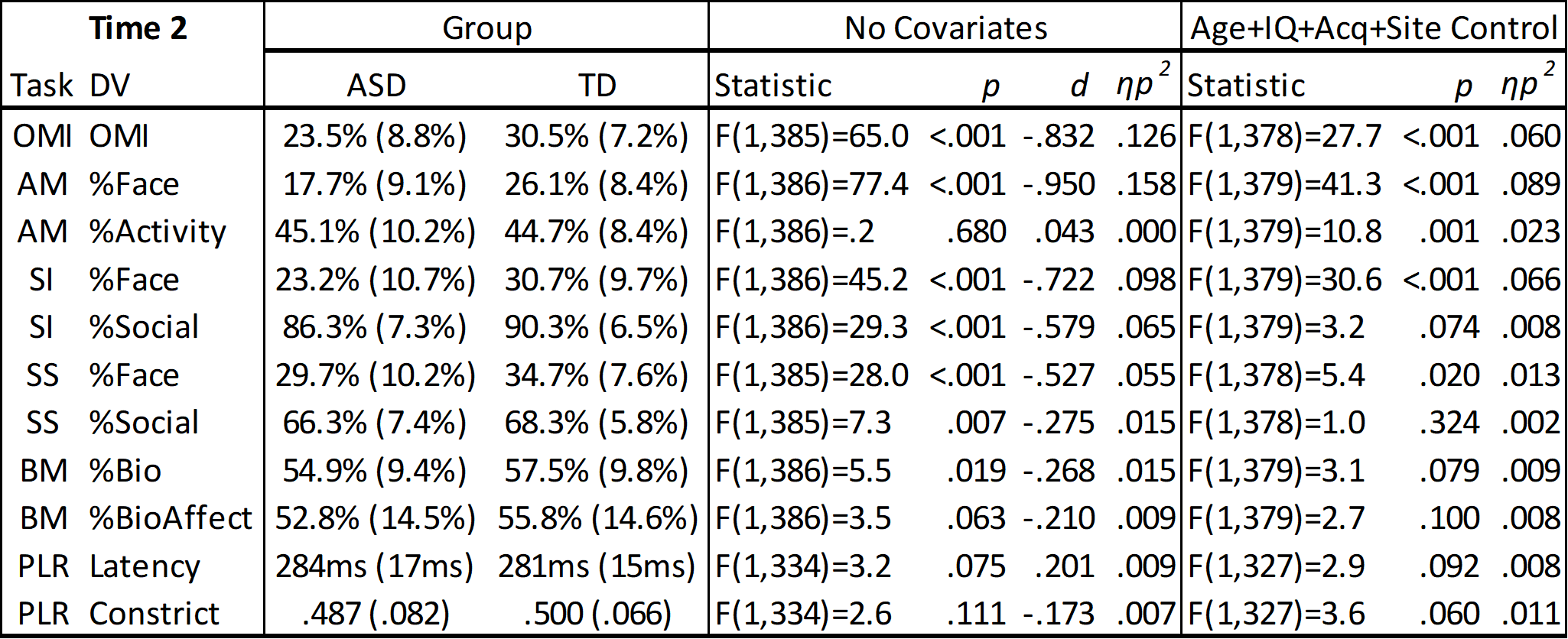
**Table Key: OMI=Oculomotor Index of Gaze to Human Faces; AM=Activity Monitoring; SI=Social Interactive; SS=Static Scenes; BM=Biological Motion Preference; PLR=Pupilary Light Reflex; DV = dependent variable; Age = participant age; IQ = Full Scale IQ; Acq = %Valid data collection rate; Site = data collection site.

## Clinical Correlations

#### **Table S13a. Spearman’s Correlations between ET and child behaviors in the ASD group at T1.** Underlined correlations are significant at *p*<.05 even after controlling for Age, Full Scale IQ, and %Valid Data.


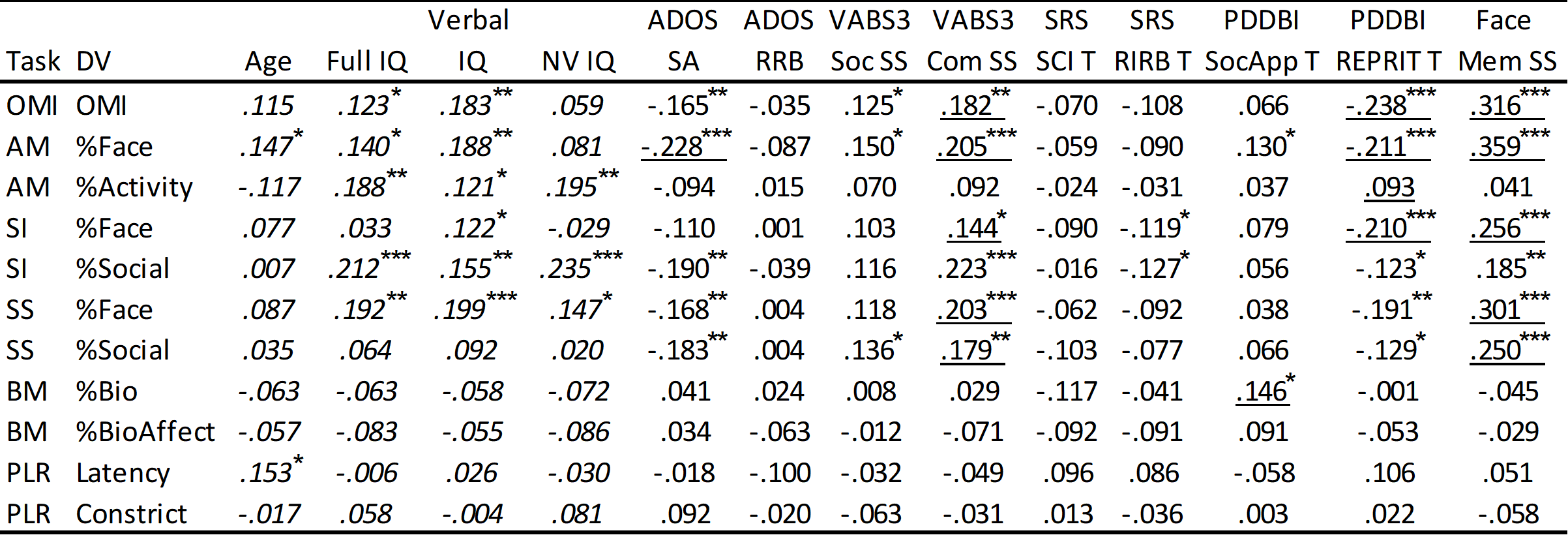


Table Key: OMI=Oculomotor Index of Gaze to Human Faces; AM=Activity Monitoring; SI=Social Interactive; SS=Static Scenes; BM=Biological Motion Preference; PLR=Pupilary Light Reflex; Full IQ = DAS Full Scale IQ; NV IQ = DAS Nonverbal IQ; ADOS SA = Autism Diagnostic Observation Schedule social affect comparison score; ADOS RRB = restricted interests and repetitive behavior comparison score; VABS3 Soc = Vineland Adaptive Behavior Scales adaptive behavior socialization standard score; VABS3 Com = communication standard score; SRS-2 SCI = Social Responsiveness Scale social communication and interaction T-score; SRS-2 RRB = restricted interest and repetitive behavior T-score; PDDBI SocApp T = Pervasive Developmental Disorders Behavior Inventory Social Approach Behaviors T-score; PDDBI REPRIT = Repetitive, Ritualistic, and Pragmatic Problems Composite T-score; Face Mem SS = NEPSY memory for faces subtask score. ^*^*p*<.05; ^**^*p*<.01; ^***^*p*<.001. Underlined cells are significant even after controlling for Age, Full Scale IQ, and %Valid Data. *Italicized* cells cannot be controlled for these variables due to collinearity.

#### **Table S13a1. Pearson’s Correlations between ET and child behaviors in the ASD group at T1.** Underlined correlations are significant at *p*<.05 even after controlling for Age, Full Scale IQ, and %Valid Data.

Table Key: OMI=Oculomotor Index of Gaze to Human Faces; AM=Activity Monitoring; SI=Social Interactive; SS=Static Scenes; BM=Biological Motion Preference; PLR=Pupilary Light Reflex; Full IQ = DAS Full Scale IQ; NV IQ = DAS Nonverbal IQ; ADOS SA = Autism Diagnostic Observation Schedule social affect comparison score; ADOS RRB = restricted interests and repetitive behavior comparison score; VABS3 Soc = Vineland Adaptive Behavior Scales adaptive behavior socialization standard score; VABS3 Com = communication standard score; SRS-2 SCI = Social Responsiveness Scale social communication and interaction T-score; SRS-2 RRB = restricted interest and repetitive behavior T-score; PDDBI SocApp T = Pervasive Developmental Disorders Behavior Inventory Social Approach Behaviors T-score; PDDBI REPRIT = Repetitive, Ritualistic, and Pragmatic Problems Composite T-score; Face Mem SS = NEPSY memory for faces subtask score. ^*^*p*<.05; ^**^*p*<.01; ^***^*p*<.001. Underlined cells are significant even after controlling for Age, Full Scale IQ, and %Valid Data. *Italicized* cells cannot be controlled for these variables due to collinearity.

#### **Table S13a2. Kendall’s Tau Correlations between ET and child behaviors in the ASD group at T1.** Underlined correlations are significant at *p*<.05 even after controlling for Age, Full Scale IQ, and %Valid Data.

Table Key: OMI=Oculomotor Index of Gaze to Human Faces; AM=Activity Monitoring; SI=Social Interactive; SS=Static Scenes; BM=Biological Motion Preference; PLR=Pupilary Light Reflex; Full IQ = DAS Full Scale IQ; NV IQ = DAS Nonverbal IQ; ADOS SA = Autism Diagnostic Observation Schedule social affect comparison score; ADOS RRB = restricted interests and repetitive behavior comparison score; VABS3 Soc = Vineland Adaptive Behavior Scales adaptive behavior socialization standard score; VABS3 Com = communication standard score; SRS-2 SCI = Social Responsiveness Scale social communication and interaction T-score; SRS-2 RRB = restricted interest and repetitive behavior T-score; PDDBI SocApp T = Pervasive Developmental Disorders Behavior Inventory Social Approach Behaviors T-score; PDDBI REPRIT = Repetitive, Ritualistic, and Pragmatic Problems Composite T-score; Face Mem SS = NEPSY memory for faces subtask score. ^*^*p*<.05; ^**^*p*<.01; ^***^*p*<.001. Underlined cells are significant even after controlling for Age, Full Scale IQ, and %Valid Data. *Italicized* cells cannot be controlled for these variables due to collinearity.

#### **Table S13b. Spearman’s Correlations between ET and child behaviors in the TD group at T1.** Underlined correlations are significant at *p*<.05 even after controlling for Age, Full Scale IQ, and %Valid Data.
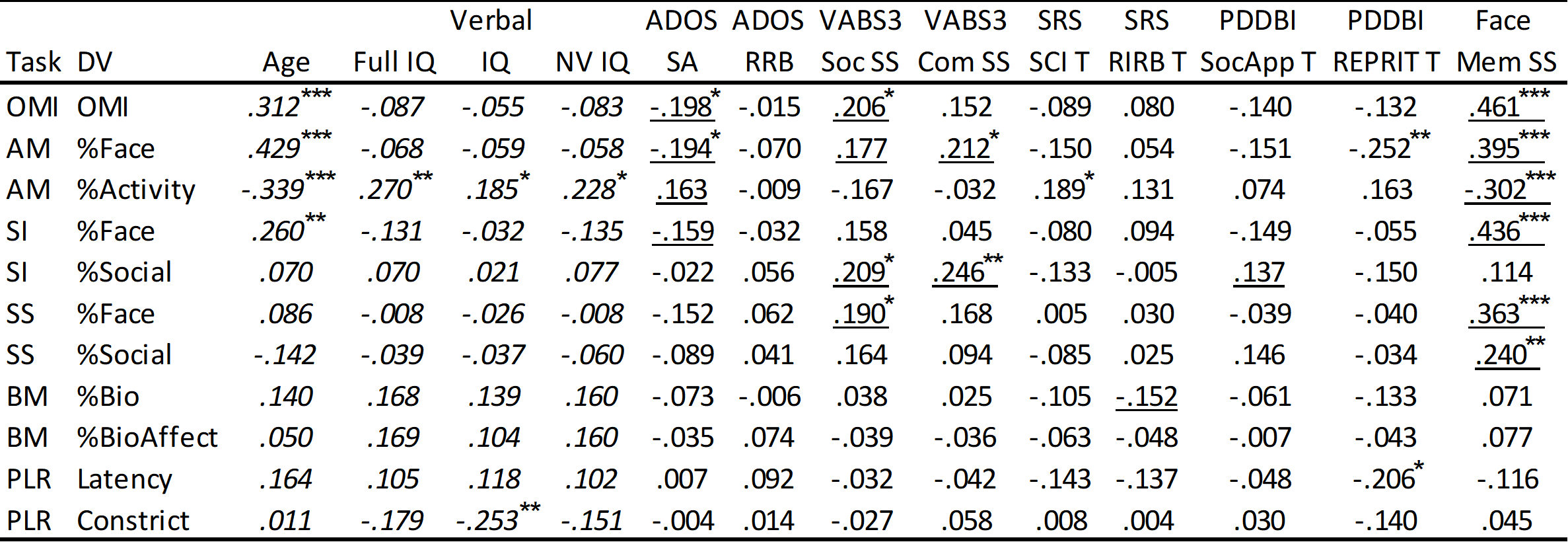


Table Key: OMI=Oculomotor Index of Gaze to Human Faces; AM=Activity Monitoring; SI=Social Interactive; SS=Static Scenes; BM=Biological Motion Preference; PLR=Pupilary Light Reflex; Full IQ = DAS Full Scale IQ; NV IQ = DAS Nonverbal IQ; ADOS SA = Autism Diagnostic Observation Schedule social affect comparison score; ADOS RRB = restricted interests and repetitive behavior comparison score; VABS3 Soc = Vineland Adaptive Behavior Scales adaptive behavior socialization standard score; VABS3 Com = communication standard score; SRS-2 SCI = Social Responsiveness Scale social communication and interaction T-score; SRS-2 RRB = restricted interest and repetitive behavior T-score; PDDBI SoccApp T = Pervasive Developmental Disorders Behavior Inventory Social Approach Behaviors T-score; PDDBI REPRIT = Repetitive, Ritualistic, and Pragmatic Problems Composite T-score; Face Mem SS = NEPSY memory for faces subtask score. ^*^*p*<.05; ^**^*p*<.01; ^***^*p*<.001. Underlined cells are significant even after controlling for Age, Full Scale IQ, and %Valid Data. *Italicized* cells cannot be controlled for these variables due to collinearity.

## Sex-specific/Sex-controlled Analyses (Preliminary)

Because male:female sex ratios were not significantly different between ASD and TD groups, examination of sex differences was not originally planned for in this set of analytics. However, exploratory analyses were conducted to ascertain whether additional control for sex-effects in the form of a simple (non-interactional) covariate (between-group analyses), or stratification of results by sex (other analyses), would lead to heterogeneity in reported results. All exploratory analyses with the exception of construct validity tests (where results are reported only for the TD group) were limited to the ASD group.

For signal acquisition and validity, analyses conducted on males only yielded the same significance pattern as the results conducted on the whole group. Analyses conducted on females only yielded no differences in acquisition or validity rates, with a noticeable absence of PLR site effects in females with ASD (X^2^=5.09, p=.322). For construct validity, all task hypotheses remained significant at comparable levels whether restricted to males, females, or encompassing the entire sample. For six-week stability, results restricted to males alone were similar (<.05 ICC deviation) to those seen for the full sample; for females, ICCs for Biomotion %Bio was lower (.425 vs. .505 in the whole group); StaticScenes %Face was lower (.568 vs. .680); PLR Latency was higher (.875 vs. .749); and all other ICCs were comparable (<.05 ICC difference). Regardless, significance patterns were unchanged from whole-group patterns when restricting to any one sex. For group discrimination, controlling for participant sex did not change the significance pattern of any reported finding. For clinical relationships, restriction to just males yielded results similar overall to those reported for the whole group; for females, while overall direction (and often effect sizes) were similar to the whole-group analyses, patterns of reported *p*-values weakened, potentially due to the smaller sample of girls in the ASD group overall.

Notwithstanding the current results, additional, concerted investigation into the effects of sex on ET biomarker performance is warranted and awaits further, rigorous, scrutiny.

# ABC-CT Manuals

**Available:** <https://medicine.yale.edu/ycci/programsprojects/autism/postersandpapers/>

- Shic, F., Naples, A., Barney, E., Chang, A., Li, B., McAllister, T., Kim, M., Hasselmo, S., Atyabi,

A., Wang, Q., Bernier, R., Dawson, G., Dziura, J., Faja, S., Jeste, S., Murias, M., Nelson, C.,

Webb, S.J., Sugar, C.A., McPartland, J., and the ABC-CT Network. (2018). **ABC-CT Data**

**Acquisition and Analytic Core ET Main Study Manual of Operations, Version 4.8**. Seattle WA.

- Naples, A., Barney, E., Chang, S.A., Li, B., McAllister, T., Kim, M., Hasselmo, S., Atyabi, A.,

Wang, Q., Bernier, R., Dawson, G., Dziura, J., Faja, S., Jeste, S., Murias, M., Nelson, C., Webb,

S.J., Sugar, C.A., McPartland, J., Shic, F., and the ABC-CT Network. (2019). **ABC-CT Data**

- **Acquisition and Analytic Core ET Main Study Acquisition Protocol, Version 5.0**. Seattle WA.
- Barney, E., Chang, S.A., Li, B., McAllister, T., Kim, M., Hasselmo, S., Atyabi, A., Wang, Q.,

Bernier, R., Dawson, G., Dziura, J., Faja, S., Jeste, S., Murias, M., Nelson, C., Webb, S.J.,

Sugar, C.A., McPartland, J., Naples, A., Shic, F., and the ABC-CT Network. (2018). **ABC-CT**

**Data Acquisition and Analytic Core ET Main Study Quality Control Manual, Version 1.3**. Seattle WA.

- Shic, F., Naples, A., Barney, E., Chang, S.A., Li, B., McAllister, T., Kim, M., Hasselmo, S.,

Atyabi, A., Wang, Q., Bernier, R., Dawson, G., Dziura, J., Faja, S., Jeste, S., Murias, M.,

Nelson, C., Webb, S.J., Sugar, C.A., McPartland, J., and the ABC-CT Network. (2018). **ABC-CT**

**Data Acquisition and Analytic Core ET Main Study Derived Results Manual, Version 1.5**. Seattle WA.

# References

1. American Psychiatric Association (2013): *Diagnostic and Statistical Manual of Mental Disorders: DSM-5.* Arlington, Va.: American Psychiatric Association.

2. Lord C, Rutter M, DiLavore PC, Risi S, Gotham K, Bishop S (2012): *Autism Diagnostic Observation Schedule: ADOS-2*. Western Psychological Services Los Angeles, CA.

3. Rutter M, LeCouteur A, Lord C (2003): Autism Diagnostic Interview-Revised (ADI-R). *West Psychol Serv Los Angel CA*.

4. Elliott CD (2007): *Differential Abilities Scale II*. San Antonio, TX: Pearson Education, Inc.

5. Gadow KD, Sprafkin J (2013): *Child and Adolescent Symptom Inventory-5 (CASI-5)*. Stonybrooke, New York: Checkmate Plus.

6. Constantino JN, Gruber CP (2012): *Social Responsiveness Scale Second Edition (SRS-2): Manual*. Western Psychological Services (WPS).

7. Cohen IL, Sudhalter V (2005): *PDD Behavior Inventory (PDDBI)*. Lutz, FL: Psychological Assessment Resources.

8. Sparrow SS, Cicchetti DV, Saulnier CA (2016): *Vineland-3: Vineland Adaptive Behavior Scales*. PsychCorp.

9. Korkman M, Kirk U, Kemp S (2007): *NEPSY-Second Edition (NEPSY-II)*. San Antonio, TX: The Psychological Corporation. Retrieved from http://scholar.google.com/scholar?q=related:VCoT37dEvCEJ:scholar.google.com/&hl=en&num=20&as_sdt=0,5

10. Shic F (2008): *Computational Methods for Eye-Tracking Analysis: Applications to Autism*. Yale University.

11. Duchowski AT (2007): *Eye Tracking Methodology: Theory and Practice*. New York: Springer-Verlag.

12. Holmqvist K, Nyström M, Andersson R, Dewhurst R, Jarodzka H, Van de Weijer J (2011): *Eye Tracking: A Comprehensive Guide to Methods and Measures*. New York: Oxford University Press Inc.

13. Fan X, Miles JH, Takahashi N, Yao G (2009): Abnormal Transient Pupillary Light Reflex in Individuals with Autism Spectrum Disorders. *J Autism Dev Disord* 39: 1499–1508.

14. Nyström P, Gredebäck G, Bölte S, Falck-Ytter T, Team E (2015): Hypersensitive pupillary light reflex in infants at risk for autism. *Mol Autism* 6: 1–6.

15. Shic F, Bradshaw J, Klin A, Scassellati B, Chawarska K (2011): Limited activity monitoring in toddlers with autism spectrum disorder. *Brain Res* 1380: 246–254.

16. Shic F, Chen G, Perlmutter M, Gisin E, Dowd A, Prince E, *et al.* (2014, May): Components of Limited Activity Monitoring in Toddlers and Children with ASD. presented at the 2014 International Meeting for Autism Research (IMFAR 2014), Atlanta, Georgia, US.

17. Del Valle Rubido M, Hollander E, McCracken JT, Shic F, Noeldeke J, Boak L, *et al.* (2020): Exploring Social Biomarkers in High-Functioning Adults with Autism and Asperger’s Versus Healthy Controls: A Cross-Sectional Analysis. *J Autism Dev Disord*. https://doi.org/10/gg2gmn

18. Kaliukhovich D, Manyakov NV, Bangerter A, Ness S, Skalkin A, Boice M, *et al.* (2019): P.507 Visual activity monitoring and social attention in autism spectrum disorder: effect of context and age. *Eur Neuropsychopharmacol* 29: S357–S358.

19. Chevallier C, Parish-Morris J, McVey A, Rump KM, Sasson NJ, Herrington JD, Schultz RT (2015): Measuring social attention and motivation in autism spectrum disorder using eye-tracking: Stimulus type matters. *Autism Res* 8: 620–628.

20. Pelphrey KA, Sasson NJ, Reznick JS, Paul G, Goldman BD, Piven J (2002): Visual scanning of faces in autism. *J Autism Dev Disord* 32: 249–261.

21. Riby DM, Hancock PJB (2008): Viewing it differently: Social scene perception in Williams syndrome and Autism. *Neuropsychologia* 46: 2855–2860.

22. Gliga T, Elsabbagh M, Andravizou A, Johnson M (2009): Faces Attract Infants’ Attention in Complex Displays. *Infancy* 14: 550–562.

23. Wass SV, Jones EJH, Gliga T, Smith TJ, Charman T, Johnson MH, *et al.* (2015): Shorter spontaneous fixation durations in infants with later emerging autism. *Sci Rep* 5: 8284–8.

24. Webb SJ, Shic F, Murias M, Sugar CA, Naples AJ, Barney E, *et al.* (2020): Biomarker Acquisition and Quality Control for Multi-Site Studies: The Autism Biomarkers Consortium for Clinical Trials. *Front Integr Neurosci* 13. https://doi.org/10.3389/fnint.2019.00071

25. Frazier TW, Strauss M, Klingemier EW, Zetzer EE, Hardan AY, Eng C, Youngstrom EA (2017): A Meta-Analysis of Gaze Differences to Social and Nonsocial Information Between Individuals With and Without Autism. *J Am Acad Child Adolesc Psychiatry* 0. https://doi.org/10.1016/j.jaac.2017.05.005

26. Johansson G (1973): Visual perception of biological motion and a model for its analysis. *Percept Psychophys* 14: 201–211.

27. Simion F, Regolin L, Bulf H (2008): A predisposition for biological motion in the newborn baby. *Proc Natl Acad Sci* 105: 809.

28. Vallortigara G, Regolin L, Marconato F (2005): Visually inexperienced chicks exhibit spontaneous preference for biological motion patterns. *PLoS Biol* 3: e208.

29. Annaz D, Remington A, Milne E, Coleman M, Campbell R, Thomas M, Swettenham J (2010): Atypical development of motion processing trajectories in children with autism. *Dev Sci Publ Online Dec 28 2009*.

30. Blake R, Turner LM, Smoski MJ, Pozdol SL, Stone WL (2003): Visual Recognition of Biological Motion Is Impaired in Children with Autism. *Psychol Sci* 14: 151–157.

31. Hubert B, Wicker B, Moore DG, Monfardini E, Duverger H, Fonséca DD, Deruelle C (2007): Brief report: recognition of emotional and non-emotional biological motion in individuals with autistic spectrum disorders. *J Autism Dev Disord* 37: 1386–1392.

32. Kaiser MD, Shiffrar M (2009): The visual perception of motion by observers with autism spectrum disorders: A review and synthesis. *Psychon Bull Rev* 16: 761–777.

33. Annaz D, Karmiloff-Smith A, Johnson MH, Thomas MSC (2009): A cross-syndrome study of the development of holistic face recognition in children with autism, Down syndrome, and Williams syndrome. *J Exp Child Psychol* 102: 456–486.

34. Kaiser MD, Delmolino L, Tanaka JW, Shiffrar M (2010): Comparison of visual sensitivity to human and object motion in autism spectrum disorder. *Autism Res*. https://doi.org/10/ffwm9r

35. Klin A, Lin D, Gorrindo P, Ramsay G, Jones W (2009): Two-year-olds with autism orient to non-social contingencies rather than biological motion. *Nature* 459: 257–261.

36. CMU Graphics Lab (2011, September 6): Carnegie Mellon University - CMU Graphics Lab - motion capture library. Retrieved September 6, 2011, from http://mocap.cs.cmu.edu/

37. Daluwatte C, Miles JH, Christ SE, Beversdorf DQ, Takahashi TN, Yao G (2012): Atypical Pupillary Light Reflex and Heart Rate Variability in Children with Autism Spectrum Disorder. *J Autism Dev Disord* 43: 1910–1925.

38. Nyström P, Gliga T, Nilsson Jobs E, Gredebäck G, Charman T, Johnson MH, *et al.* (2018): Enhanced pupillary light reflex in infancy is associated with autism diagnosis in toddlerhood [no. 1]. *Nat Commun* 9: 1678.

39. Itti L, Koch C, Niebur E (1998): A model of saliency-based visual attention for rapid scene analysis. *Pattern Anal Mach Intell IEEE Trans On* 20: 1254–1259.

40. Shic F, Scassellati B (2007): A Behavioral Analysis of Computational Models of Visual Attention. *Int J Comput Vis* 73: 159–177.

41. Shic F, Chawarska K, Lin D, Scassellati B (2007): Measuring context: The gaze patterns of children with autism evaluated from the bottom-up. *Development and Learning, 2007. ICDL IEEE 6th International Conference On* 70–75.
